# Effect of Duration and Intermittency of Rifampin on Tuberculosis Treatment Outcomes: A Systematic Review and Meta-Analysis

**DOI:** 10.1371/journal.pmed.1000146

**Published:** 2009-09-15

**Authors:** Dick Menzies, Andrea Benedetti, Anita Paydar, Ian Martin, Sarah Royce, Madhukar Pai, Andrew Vernon, Christian Lienhardt, William Burman

**Affiliations:** 1 Respiratory and Epidemiology Clinical Research Unit, Montreal Chest Institute & Department of Epidemiology, Biostatistics & Occupational Health, McGill University, Montreal, Canada; 2 University of California at San Francisco, San Francisco, California, United States of America; 3 Centers for Disease Control and Prevention, Atlanta, Georgia, United States of America; 4 International Union against Tuberculosis and Lung Diseases, and Institut de Recherche pour le Développement, Paris, France; 5 Denver Public Health, Denver, Colorado, United States of America; Harvard School of Public Health, United States of America

## Abstract

**Background:**

Treatment regimens for active tuberculosis (TB) that are intermittent, or use rifampin during only the initial phase, offer practical advantages, but their efficacy has been questioned. We conducted a systematic review of treatment regimens for active TB, to assess the effect of duration and intermittency of rifampin use on TB treatment outcomes.

**Methods and Findings:**

PubMed, Embase, and the Cochrane CENTRAL database for clinical trials were searched for randomized controlled trials, published in English, French, or Spanish, between 1965 and June 2008. Selected studies utilized standardized treatment with rifampin-containing regimens. Studies reported bacteriologically confirmed failure and/or relapse in previously untreated patients with bacteriologically confirmed pulmonary TB. Pooled cumulative incidences of treatment outcomes and association with risk factors were computed with stratified random effects meta-analyses. Meta-regression was performed using a negative binomial regression model. A total of 57 trials with 312 arms and 21,472 participants were included in the analysis. Regimens utilizing rifampin only for the first 1–2 mo had significantly higher rates of failure, relapse, and acquired drug resistance, as compared to regimens that used rifampin for 6 mo. This was particularly evident when there was initial drug resistance to isoniazid, streptomycin, or both. On the other hand, there was little evidence of difference in failure or relapse with daily or intermittent schedules of treatment administration, although there was insufficient published evidence of the efficacy of twice-weekly rifampin administration throughout therapy.

**Conclusions:**

TB treatment outcomes were significantly worse with shorter duration of rifampin, or with initial drug resistance to isoniazid and/or streptomycin. Treatment outcomes were similar with all intermittent schedules evaluated, but there is insufficient evidence to support administration of treatment twice weekly throughout therapy.

*Please see later in the article for the Editors' Summary*

## Abstract 2

In a systematic review of randomized controlled trials on tuberculosis treatment, Dick Menzies and colleagues find shorter courses of rifampin to be associated with poorer treatment outcomes.

## Editors' Summary

**Background:**

Tuberculosis—a contagious infection, usually of the lungs—kills nearly two million people annually. It is caused by *Mycobacterium tuberculosis*, bacteria that are spread in airborne droplets when people with tuberculosis cough. Most people infected with *M. tuberculosis* do not become ill—their immune system contains the infection. However, the bacteria remain dormant within the body and can cause tuberculosis years later if immunity declines because of, for example, infection with HIV (the virus that causes AIDS). The symptoms of tuberculosis include a persistent cough, weight loss and night sweats. The disease can usually be cured by taking several powerful antibiotics regularly for several months although drug-resistant tuberculosis is increasingly widespread. The standardized drug regimen recommended by the World Health Organization (WHO) for previously uninfected patients consists of an initial treatment phase, in which rifampin, isoniazid, ethambutol, and pyrazinamide are taken daily or thrice weekly for 2 months, and a continuation phase, in which two antibiotics are taken for a further 4–6 months.

**Why Was This Study Done?:**

Resistance to rifampicin, which can develop if this drug is not taken regularly, is associated with poor treatment outcomes, particularly in patients infected with isoniazid-resistant *M. tuberculosis*. WHO recommends, therefore, that health-care workers watch patients take all their doses of rifampicin (“directly observed therapy”). Treatment regimens for tuberculosis that use rifampicin only during the initial phase and/or give rifampicin several times a week (intermittently) rather than daily would make direct observation of treatment much easier but are such regimens effective? In this study (which, together with two similar studies, was commissioned by WHO to provide the evidence needed for a revision of their treatment guidelines), the researchers undertook a systematic review (a search using specific criteria to identify relevant research studies, which are then appraised and analyzed according to an explicit protocol) and a meta-analysis (a statistical approach that pools the results of several studies) of published trials of various rifampicin-containing regimens for the treatment of tuberculosis in previously untreated patients.

**What Did the Researchers Do and Find?:**

The researchers identified 57 randomized controlled trials (studies in which groups of patients are randomly assigned to different interventions) that reported the treatment failure and/or relapse rates (determined by seeing whether *M. tuberculosis* could be grown from sputum brought up from the lungs by coughing, so-called bacteriologically confirmed tuberculosis) associated with various rifampicin-containing treatment regimens. In their statistical analysis of the results of these trials (which involved more than 21,000 previously untreated patients), the researchers found that regimens that used rifampicin during only the first 1–2 months of treatment had higher rates of failure, relapse, and acquired drug resistance than regimens that used rifampicin for 6 months. Indeed, relapse rates decreased with the duration of rifampicin treatment up to 8 months of treatment. Furthermore, outcomes were particularly bad with regimens that included rifampicin during only the first 1–2 months of treatment if there was initial resistance to isoniazid and/or streptomycin (another antibiotic). Outcomes were similar, however, in regimens in which rifampicin was given daily throughout treatment, daily during the initial phase then twice or thrice weekly, or thrice weekly throughout treatment; insufficient evidence was available to evaluate the efficacy of regimens in which rifampicin was given twice weekly throughout treatment.

**What Do These Findings Mean?:**

These findings suggest that tuberculosis treatment regimens for previously untreated patients who use rifampicin during only the first two months of treatment should be phased out and replaced by regimens that use rifampicin for 6 months, particularly in settings where there is likely to be resistance to isoniazid and/or streptomycin. This recommendation will be made in the planned 2009 revision of the WHO tuberculosis treatment guidelines. In addition, these findings suggest that giving rifampicin thrice weekly is as effective as giving it daily during the initial phase or throughout treatment. Importantly, these findings also indicate that more trials are needed to investigate other dosing schedules, to determine the optimal duration of treatment, and to determine the best way to manage patients infected with isoniazid-resistant bacteria. Finally, since very few of the trials identified in the systematic review included HIV-positive participants, trials designed to test drug regimens in people infected with both HIV and *M. tuberculosis* are urgently needed to reduce global deaths from tuberculosis.

**Additional Information:**

Please access these Web sites via the online version of this summary at http://dx.doi.org/10.1371/journal.pmed.1000146.

- The results of another WHO-commissioned study into the treatment of tuberculosis are presented in a separate *PLoS Medicine* Research Article by Menzies et al. (Menzies D, Benedetti A, Paydar A, Royce S, Pai M, et al. (2009) Standardized Treatment of Active Tuberculosis in Patients with Previous Treatment and/or with Mono-resistance to Isoniazid: A Systematic Review and Meta-analysis. PLoS Med 6(9): e1000150. doi:10.1371/journal.pmed.1000150)
- The US National Institute of Allergy and Infectious Diseases provides information on all aspects of tuberculosis
- The US Centers for Disease Control and Prevention provide several facts sheets and other information resources about tuberculosis
- The American Thoracic Society, US Centers for Disease Control and Prevention, and Infectious Diseases Society of America have published guidelines on TB treatment
- The 2003 (2004 revision) WHO guidelines for national programs for the treatment of tuberculosis are available; WHO also provides information on efforts to reduce the global burden of tuberculosis (in several languages) and its 2009 annual report on global control of tuberculosis describes the current situation (key points are available in several languages)
- The WHO publishes guidelines on TB treatment

## Introduction

When rifampin was first introduced, it held the promise of exceptional potency as an agent for treatment of *Mycobacterium tuberculosis* (the cause of tuberculosis [TB]). A series of randomized trials, most conducted 20–35 y ago, established that rifampin-containing regimens could achieve high cure rates with as few as 6 mo of therapy, even when given intermittently [1]. These trials ushered in the modern era of short-course chemotherapy and established the scientific rationale for the standardized regimens currently recommended by the World Health Organization (WHO) [2]. WHO recommends direct observation of all doses of rifampin to prevent rifampin resistance, which is associated with much worse treatment outcomes, especially when combined with isoniazid resistance as multi-drug resistance (MDR) [3],[4]. This direct observation is facilitated by shorter duration of rifampin, and/or by intermittent dosing schedules.

However, the frequency of all forms of drug resistance has steadily increased in many countries over the last 20 y [5]–[7]. The effectiveness of current empiric regimens in treating patients with unrecognized initial drug resistance of any form, including isoniazid mono-resistance, is unclear since this was not a focus of earlier trials. In particular, the adequacy of regimens that use rifampin only in the initial phase, or are intermittent throughout, may be questionable in settings with increasing drug resistance. In addition, the individual trials that form the scientific basis for current therapy were mostly small trials with limited power to evaluate the duration and intermittency of rifampin use. Hence meta-analysis of these trials can allow evaluation of factors that may have a small but clinically relevant effect on treatment outcomes.

In light of these uncertainties, we have conducted a systematic review of treatment regimens for active TB, to provide a basis for recommendations for revised treatment guidelines.

### Review Questions

Our systematic review aimed to address two specific questions:

1. What are the rates of treatment failure, relapse, and acquired drug resistance if rifampin is given only in the initial intensive phase (the first 1–2 mo), compared to longer duration?
2. What are the rates of treatment failure, relapse, and acquired drug resistance with different dose administration schedules of therapy?

## Methods

### Search Strategy

We searched three electronic databases—PubMed, Embase, and the Cochrane CENTRAL database—for studies of treatment of active TB (i.e., disease). The search was restricted to randomized controlled trials published in English, French, and Spanish from 1965 up to June 2008. Our keywords included tuberculosis or TB, treatment or therapy, failure or relapse, or drug resistance. To identify additional relevant articles, we searched reference lists of identified original articles, recent systematic reviews [8]–[11], a review of all the British Medical Research Council trials [12], recent treatment guidelines [13],[14], and texts [15],[16].

### Study Selection

We included original reports of randomized controlled trials that reported treatment outcomes of bacteriologically confirmed failure and/or relapse. In selected trials, all patients had active pulmonary TB that was bacteriologically confirmed by AFB smear microscopy and/or mycobacterial culture, and had not been previously treated (i.e., were new cases). Treatment was standardized and included at least isoniazid and rifampin. We excluded trials or arms that included rifapentine or rifabutin therapy or non-drug therapy (e.g., immunotherapeutic vaccines). We also excluded trials, or arms, that involved once-weekly or mono-drug therapy, as these are now considered inadequate [13],[14].

The selection of articles for review was done independently by two investigators in three stages: titles alone, followed by abstracts, and then full text articles. Decisions were compared and disagreements about study selection were resolved by consensus or by involving a third reviewer.

### Data Extraction and Quality Assessment

We reviewed all selected studies using standardized forms to extract data about patient population and characteristics, treatment regimens, pretreatment drug-susceptibility testing, supervision of treatment, funding source, and number of patients who started treatment, defaulted or were otherwise lost, died, failed, or relapsed. Two reviewers extracted the data, with disagreements resolved by consensus.

We restricted the studies reviewed to randomized trials with bacteriologic confirmation of initial diagnosis, failure, and/or relapse—considered high-quality methods. Included studies were considered of high quality if less than 10% of patients refused therapy, dropped out, moved away, or were otherwise unaccounted for during therapy. In addition, randomized trials were considered high quality if they used an allocation concealment approach such as central randomization, numbered opaque sealed envelopes, sealed envelopes from a closed bag, numbered or coded bottles or containers, or if treatments were assigned by a central pharmacy.

### Outcomes

In line with internationally accepted definitions [17], treatment failure was defined as sputum smears and/or cultures that were consistently positive or requiring treatment at the end of therapy (if less than 5 mo) or after at least 5 mo of therapy. Relapse was defined as recurrence of positive smears and/or cultures that required therapy after completion of treatment with apparent cure. Initial drug resistance was defined as pretreatment resistance in patients without a history of previous treatment, and categorized as pan-sensitive, isoniazid resistant, streptomycin resistant, or resistant to both—termed poly-drug resistance. Patients with initial rifampin resistance, including MDR, were excluded from analysis, if identified in the published report. Acquired drug resistance was defined as new or additional resistance to one or more of the TB drugs received, among failures or relapses.

### Data Synthesis and Analysis

We were interested in understanding the efficacy of different regimens in preventing failure, relapse, and acquired drug resistance—end-points with objective microbiological definitions that were consistent across trials. Therefore, we used a per-protocol analysis, excluding patients who did not complete therapy because they developed serious adverse reactions, died, defaulted, dropped out, or were otherwise not accounted for.

We restricted our first analysis to those trials within which regimens differed by the duration of rifampin, or dosing schedule (intermittency), but were otherwise comparable. To increase the number of trials with head-to-head comparisons analyzed, regimens were considered comparable even if they differed by ethambutol or thiacetazone. For each trial with head-to-head comparisons, we calculated cumulative incidence of failure, relapse, and acquired drug resistance, and the Mantel-Haenszel pooled difference in cumulative incidence and 95% confidence interval (CI) for each comparison [18]. One advantage of this method is that 0-cell corrections are not necessary to calculate the MH pooled risk difference [18]. We assessed heterogeneity of risk differences for each comparison, by estimating the I^2^ statistic and associated 95% CIs [19]. For this calculation, studies in which both arms had no events were corrected by 0.5.

Because few trials with head-to-head comparisons were identified, in our second analysis we pooled results across all trials, effectively treating each arm within each trial as an independent cohort. For the across-trial analysis, we used a random effects meta-analysis to estimate the overall pooled estimates of cumulative incidence and 95% CI of failure, relapse, and acquired drug resistance using Proc Nlmixed in SAS (SAS Institute, Carey, NC, USA) [20]. We used the exact binomial likelihood approach [20], which uses a binomial distribution to approximate the distribution of the outcome of interest. This approach accounts for study size and includes a random effect to account for between-study heterogeneity. When proportions are the outcome measure, this approach has been demonstrated to produce less-biased estimates of the pooled effect and the between-study variability [20]. We assessed heterogeneity of proportions of participants with outcomes of interest, within subgroups defined by covariates of interest by estimating the I^2^ statistic and associated 95% CIs [19]. To calculate I^2^, 0-cells were corrected by 0.5.

To minimize heterogeneity, we performed subgroup analyses stratified by predefined covariates of interest. These included duration and dosing schedule of rifampin, initial drug resistance, use of pyrazinamide or streptomycin, and number of drugs to which the organism was susceptible used in the initial or continuation phase (the initial intensive phase was defined as the initial period when more drugs were used—usually the first 1–2 mo—while the continuation phase was the remainder of therapy). We also examined the effect of supervision of therapy (i.e. directly observed therapy [DOT]), proportion that were smear positive, and default or other losses during treatment phase follow-up.

Finally, meta-regression was used to estimate the effect of the treatment factors of interest, after adjustment for other potentially confounding patient and treatment covariates. Because the outcomes we were pooling were proportions rather than odds ratios, and because these proportions were usually small, we performed meta-regression using the Poisson model [21] that allowed for overdispersion (i.e., negative binomial regression). In this meta-regression, each arm in each study was the unit of analysis, cumulative incidence of TB treatment outcomes was the dependent variable, and TB treatment characteristics were the independent variables. An offset was used to account for size of study. In this approach, residual heterogeneity between studies is accounted for in the dispersion parameter. As such we interpreted the dispersion parameter as indicating there was no remaining unexplained heterogeneity if the value was not significantly different from zero and as minimal heterogeneity if the value was less than 1.0 [22]. Effect estimates of the meta-regression model were interpreted as adjusted incidence rate ratios (IRR) [21]. We tested the significance of each factor in the models using the likelihood ratio test. Two models were used. The first included rifampin duration, intermittent schedule, use of pyrazinamide and streptomycin, number of drugs in initial or continuation phases to which organisms were susceptible, length of follow-up after end of treatment (for relapse and acquired drug resistance), directly observed therapy (DOT), and non-completion of therapy because of protocol violations, patient refusal, default, moved, or lost. The second model included initial drug resistance with all the same factors, except the number of sensitive drugs in initial or continuation phases—which could not be included because of substantial co-linearity between these factors and drug resistance.

## Results

### Description of Included Trials

**Figure 1:**
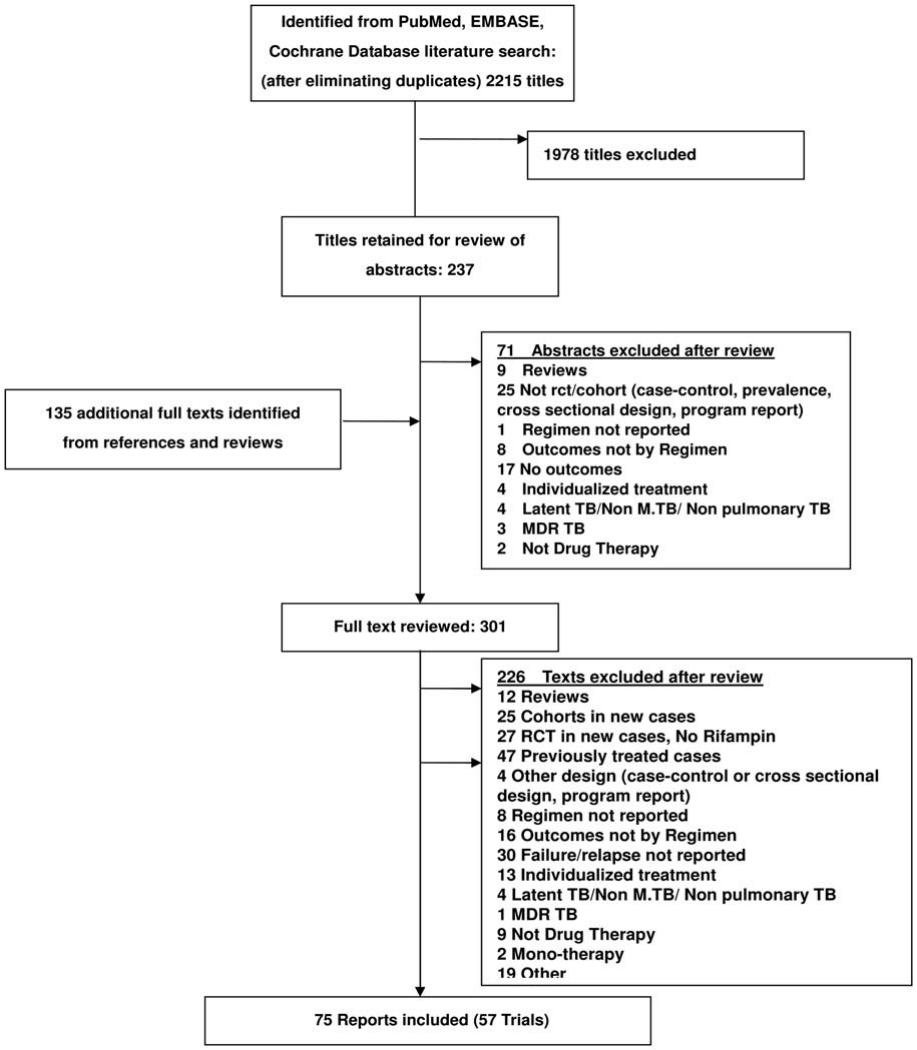
Summary of literature search and study selection.

As seen in Figure 1, 2,215 citations were identified from the search of the three electronic databases. Of these, 237 were retained for abstract review and 166 for full text review. An additional 135 full texts were identified from the search of the references of the original articles and other sources. After full text review, 75 papers describing 57 randomized trials were selected for this analysis. These trials, summarized in Appendix Table 1 in Text S1 [23]–[94], included a total of 21,472 participants in 312 different treatment arms. Of these, 612 patients died and 2,775 dropped out, were lost, or were otherwise not accounted for. This left 18,701 analyzable participants in all trials. Failure was analyzed for 18,085 participants in 56 studies; relapse was reported for 15,558 participants in 53 trials, while acquired drug resistance was reported for 11,400 participants in 39 trials (Table 1). All trials involved adults, and in 42 all patients were smear positive, although cultures were used to define study end-points in all but three trials [26],[90],[94]. Only four trials included HIV-infected persons, with a total of 522 HIV-infected participants. Rifampin dose was not stated in two trials [62],[94], was 450 mg/d in three trials with a total of 618 patients [76],[89],[95], and was 600 mg/d (or 10–12 mg/kg) in all the remaining studies. In 30 trials (52%) all treatment doses were supervised, and in 29 (51%) less than 10% refused, defaulted, or were lost during treatment. Randomization was described in 41 trials, and was adequate in 40, but not described in 16 trials. Median post-treatment follow-up was 24 mo (interquartile range: 18–30).

**Table 1:** Summary of studies reviewed (all randomized controlled trials with rifampin-containing regimens in new cases).

Characteristic	By Study		By Arm	
	*N*	%	*N*	%
**Language of publication**				
English	54	95	299	96
French	3	5	14	4
**Year when study began**				
1969–1979	26	45	178	56
1980–1989	16	28	104	33
1990–1999	13	24	26	9
2000–Present	2	3	5	2
**Sponsorship**				
Public	56	98	311	99
Corporate	1	2	2	1
**Measured failure**				
Yes	56	98	311	99
No	1	2	2	1
**Measured relapse**				
Yes	53	93	302	97
No	4	7	11	3
**Measured acquired drug resistance**				
Yes	39	69	264	84
No	18	31	49	16
**Quality in treatment**				
High (≤10% dropout/lost)	29	51	182	58
Poor (>10% dropout/lost)	28	49	131	42
**Quality in follow-up**				
High (≤10% lost)	40	67	217	72
Poor (>10% lost)	17	33	85	28
**Quality of randomization**				
Adequate	39	70	181	58
Not stated or inadequate	18	30	132	42
**Blinding**				
Single or double	4	7	14	4
None	53	93	299	96
**Supervision of rifampin**				
None/partial	27	47	49	16
All doses	30	53	264	84
**Isoniazid**				
Not used	0	0	1	1
Used	57	100	312	99
**Pyrazinamide**				
Not used	11	19	61	19
Used	46	81	252	81
**Streptomycin**				
Not used	28	49	101	32
Used	29	51	212	68
**Use of second line drugs**				
None	55	99	312	99
Yes – (1 drug)	2	1	2	1

Parameters Varied Within Studies – Summary by Arm Only	*N*	%
**Number of drugs used to which strains sensitive**		
*Initial intensive phase*		
1	2	1
2	68	23
3	153	52
4 or more	71	24
*Continuation phase*		
0 or 1	70	24
2	144	50
3 or more	74	26
**Drug sensitivity**		
Unknown (Mix)	19	6
Pan-sensitive	128	41
INH resistance	68	22
Streptomycin	54	17
Poly-drug resistance	44	14
**Rifampin use and duration**		
1–2 mo	72	23
3–4 mo	42	13
5–7 mo	178	57
8+ mo	21	7
**Schedule of administration**		
Daily throughout	161	51
Daily then thrice weekly	35	11
Daily then twice weekly	45	15
Thrice weekly throughout	70	22
Twice weekly throughout	2	1

### Results of Head-to-Head Comparisons

As seen in Table 2, in the studies with head-to-head comparisons, rates of failure (Table 2) were significantly higher in patients who received only 1–2 mo of rifampin than in patients who received longer durations of rifampin. Rates of relapse (Table 3) were progressively lower with longer duration of rifampin up to 8 mo or more. In the only trial which compared four different durations of rifampin (6 versus 9, 12, and 18 mo), all outcomes were the same with the three longer regimens [54],[86]. Acquired drug resistance (Table 4) was not associated with shorter duration of rifampin, but this outcome was uncommon and reported in fewer trials—limiting power. All five of the studies with head-to-head comparisons of intermittent schedules compared different regimens and schedules, so results could not be pooled (Table 5).

**Table 2:** Pooled risk differences from direct head-to-head comparisons of rifampin duration or intermittent schedules in otherwise comparable regimens: Failure.

Ref	Regimens^a^	Fail	Non	Fail	Non	Risk Difference
***2 versus 3–4 mo***		**2 mo**		**3–4 mo**		
[84],[110]	2SHRZ/2HZ	1	123	1	248	0.4%
	2SHRZ/2HR					
[76],[93]	2SHRZ	0	84	0	81	0
	3SHRZ					
Pooled risk difference (95% CI)						0.3% (0.9% to 1.4%)
Overall I^2^ (95% CI)						0 (− , −)
***2 versus 6 mo***		**2 mo**		**6 mo**		
[43],[111]	2SHRZ/4HZ	3	206	0	212	1.4%
	SHRZ/4HR					
[26]	2EHRZ/6HE	41	771	12	371	1.8%
	2EHRZ/4HR					
[112]	2HRZE/4[HRZ]_2_	3	41	3	51	1.3%
	2HRZE/4[HZE]_2_					
Pooled risk difference (95% CI)						1.7% (0% to 3.4%)
Overall I^2^ (95% CI)						0 (0 to 0.73)
***4 versus 6 mo***		**4 mo**		**6 mo**		
[37]–[39]	2SHRZ/2HR	0	91	1	89	−1.1%
	2SHRZ/4HR					
[37]–[39]	2SHRZ/2HRZ	0	91	0	89	0%
	2SHRZ/4HRZ					
[63]	2SHRZ/2HR/4H	4	564	1	518	0.5%
	2SHRZ/4HR					
[87]	2HRZ/2HR	0	59	1	53	−2.0%
	2HRZ/4[HR]_3_					
[90]	2HRZE/2HR	0	33	3	64	−4.5%
	2HRZE/4HR					
[91]	2SHRZ/2HR	0	79	0	83	0%
	2SHRZ/4HR					
[91]	2SHRZ/2HRZ	0	83	0	87	0%
	2SHRZ/4HRZ					
Pooled risk difference (95% CI)						−0.1% (−0.7% to 0.4%)
Overall I^2^ (95% CI)						0.10 (0 to 0.7)
***6 versus 9+ mo***		**6 mo**		**9+ mo**		
[48]	6[SHRZ]_3_	1	67	4	50	−5.9%
	8[SHRZ]_3_					
[72]	2HRZE/4[HR]_2_	5	129	4	131	0.8%
	2HRZE/10[HR]_2_					
[85]	2HRZE/4HR	1	41	0	37	2.4%
	2HRZE/7HR					
[54],[55]	2EHR/4HR	1	116	0	282	0.9%
	2EHR/7,10,16HR					
[54],[55]	2SHR/4HR	0	74	0	213	0%
	2SHR/7,10,16HR					
Pooled risk difference (95% CI)						−0.2% (−1.9% to 1.5%)
Overall I^2^ (95% CI)						0 (0 to 0.75)

Seventeen head-to-head comparisons of rifampin duration. For one study [54],[55], results with 9-, 12-, and 18-mo regimens were the same so they were combined.

a Regimen abbreviations: H, isoniazid; R, rifampin; Z, pyrazinamide; E, ethambutol; S, streptomycin.

Letters to left of “/” indicate regimen in initial intensive phase; letters to right of “/” indicate regimen in continuation phase. First number signifies the months of initial phase of treatment and the second number signifies the months of continuation phase treatment. [ ] indicates intermittent therapy; subscript number after [ ] indicates number of doses per week.

**Table 3:** Pooled risk differences from direct head-to-head comparisons of rifampin duration or intermittent schedules in otherwise comparable regimens: Relapse.

Ref	Regimens^a^	Relapse	Non	Relapse	Non	Risk Difference
***2 versus 3–4 mo***		**2 mo**		**3–4 mo**		
[84],[110]	2SHRZ/2HZ	38	78	30	200	19.7%
	2SHRZ/2HRZ					
[76]	2SHRZ	20	64	8	73	13.9%
	3SHRZ					
Pooled risk difference (95% CI)						17.7% (10.3% to 25%
Overall I^2^ (95% CI)						0 (− , −)
***2 versus 6 mo***		**2 mo**		**6 mo**		
[43],[113]	2SHRZ/4HZ	13	168	6	171	3.8%
	2SHRZ/4HR					
[26]	2EHRZ/6HE	57	344	6	236	11.7%
	2EHRZ/4HR					
[112]	2HRZE/4[HRZ]_2_	20	21	6	45	37%
	2HRZE/4[HZE]_2_					
Pooled risk difference (95% CI)						11.2% (8.1% to 14.3%)
Overall I^2^ (95% CI)						0.88 (0.66 to 0.96)
***4 versus 6 mo***		**4 mo**		**6 mo**		
[37]–[39]	2SHRZ/2HR	7	80	2	83	5.7%
	2SHRZ/4HR					
[37]–[39]	2SHRZ/2HRZ	10	79	0	82	11.2%
	2SHRZ/4HRZ					
[63]	2SHRZ/2HR/4H	38	526	23	495	2.3%
	2SHRZ/4HR					
[87]	2HRZ/2HR	0	57	0	52	0%
	2HRZ/4[HR]_3_					
[90]	2HRZE/2HR	3	30	6	58	−0.3%
	2HRZE/4HR					
[91]	2SHRZ/2HR	4	70	1	79	4.2%
	2SHRZ/4HR					
[91]	2SHRZ/2HRZ	8	72	0	84	10.0%
	2SHRZ/4HRZ					
Pooled risk difference (95% CI)						6.9% (3.7% to 10.0%)
Overall I^2^ (95% CI)						0.59 (0.05 to 0.82)
***6 vs. 9+ mo***		**6 mo**		**9+ mo**		
[48]	6[SHRZ]_3_	12	44	2	40	17%
	8[SHRZ]_3_					
[72]	2HRZE/4[HR]_2_	9	59	1	53	11.4%
	2HRZE/10[HR]_2_					
[74]	2HRZ/4HR	10	375	6	225	0%
	2HRZ/7HR					
[85]	2HRZE/4HR	1	16	1	14	−0.8%
	2HRZE/7HR					
[54],[55]	2EHR/4HR	6	96	0	213	5.9%
	2EHR/7,10,16HR					
[54],[55]	2SHR/4HR	2	52	2	160	2.5%
	2SHR/7,10,16HR					
Pooled risk difference (95% CI)						4.0% (1.8% to 6.2%)
Overall I^2^ (95% CI)						0.65 (0.18 to 0.86)

Eighteen head-to-head comparisons of rifampin duration. For one study [54],[55], results with 9-, 12-, and 18-mo regimens were the same so they were combined.

a Regimen abbreviations: H, isoniazid; R, rifampin; Z, pyrazinamide; E, ethambutol; S, streptomycin.

Letters to left of “/” indicate regimen in initial intensive phase; letters to right of “/” indicate regimen in continuation phase. First number signifies the months of initial phase of treatment and the second number signifies the months of continuation phase treatment. [ ] indicates intermittent therapy; subscript number after [ ] indicates number of doses per week.

**Table 4:** Pooled risk differences from direct head-to-head comparisons of rifampin duration or intermittent schedules in otherwise comparable regimens: Acquired drug resistance.

Ref	Regimens^a^	ADR	Non	ADR	Non	Risk Difference
***2 versus 4 mo***		**2 mo**		**4 mo**		
[76]	2SHRZ	0	84	0	81	0
	3SHRZ					
Pooled risk difference (95% CI)						0%
Overall I^2^ (95% CI)						0 (– , –)
***2 versus 6 mo***		**2 mo**		**6 mo**		
[43],[114]	2SHRZ/4HZ	3	206	2	210	0.5%
	2SHRZ/4HR					
Pooled risk difference (95% CI)						0.5% (−1.5% to 2.5%)
Overall I^2^ (95% CI)						0 (– , –)
***4 versus 6 mo***		**4 mo**		**6 mo**		
[37]–[39]	2SHRZ/2HR	1	90	1	89	0%
	2SHRZ/4HR					
[37]–[39]	2SHRZ/2HRZ	0	91	0	89	0%
	2SHRZ/4HRZ					
[63]	2SHRZ/2HR/4HR	5	563	1	518	0.7%
	2SHRZ/4HR					
[91]	2SHRZ/2HR	0	79	0	83	0%
	2SHRZ/4HR					
[91]	2SHRZ/2HRZ	0	83	0	87	0%
	2SHRZ/4HRZ					
Pooled risk difference (95% CI)						0.4% (−0.2% to 1.0%)
Overall I^2^ (95% CI)						0 (0 to 0.74)
***6 versus 9 mo***		**6 mo**		**9 mo**		
[48]	6[SHRZ]_3_	2	66	4	50	−4.5%
	8[SHRZ]_3_					
[85]	2HRZE/4HR	2	40	2	35	−0.6%
	2HRZE/7HR					
[54],[55]	2EHR/4HR	0	117	0	282	0%
	2EHR/7,10,16HR					
[54],[55]	2SHR/4HR	0	74	0	213	0%
	2SHR/7,10,16HR					
Pooled risk difference (95% CI)						−0.8% (−2.4% to 0.9%)
Overall I^2^ (95% CI)						0 (0 to 0.77)

Eleven head-to-head comparisons of rifampin duration. For one study [54],[55]—results with 9-, 12-, and 18-mo regimens were the same—so they were combined.

a Regimen abbreviations: H, isoniazid; R, rifampin; Z, pyrazinamide; E, ethambutol; S, streptomycin.

Letters to left of “/” indicate regimen in initial intensive phase; letters to right of “/” indicate regimen in continuation phase. First number signifies the months of initial phase of treatment and the second number signifies the months of continuation phase treatment. [ ] indicates intermittent therapy; subscript number after [ ] indicates number of doses per week.

**Table 5:** Pooled risk differences from direct head-to-head comparisons of rifampin duration or intermittent schedules otherwise comparable regimens.

Reference	Drug Resistance Patterns	Treatment Regimens[Table-fn nt111] a	Treated (*N*)	Failed (*N*)	Relapsed (*N*)^b^	ADR (*N*)^b^
[115],[116]	Pan-sensitive	2HRE/4[HR]_2_	93	0	16	0
		2HRE/4[HRE]_2_	96	0	6	0
		6HRE	98	0	12	0
[28],[40],[41]	Pan-sensitive, STREP, INH, PDR	6HRZE	199	0	6	0
		6[HRZE]_3_	199	1	8	0
[26]	Pan-sensitive plus all forms of resistance except MDR	2EHRZ/6HE	402	18	1	n/a
		2[EHRZ]_3_/6HE	410	22	1	n/a
[87]	Pan-sensitive	2HRZ/2HR	158	0	0	n/a
		2HRZ/2[HR]_3_	102	0	1	n/a
[117]	Pan-sensitive plus all forms of resistance except MDR	3[HRZE]_5_/3.5[HR]_5_	39	0	n/a	n/a
		6.5HRZ (with FDC)	67	0	n/a	n/a

Treatment outcomes are from five studies with direct head-to head-comparisons of intermittency schedules (and otherwise comparable regimens). Meta-analysis not done, as no schedules were the same.

a Regimen abbreviations: H, isoniazid; R, rifampin; Z, pyrazinamide; E, ethambutol; S, streptomycin.

Letters to left of “/” indicate regimen in initial intensive phase; letters to right of “/” indicate regimen in continuation phase. First number signifies the months of initial phase of treatment and the second number signifies the months of continuation phase treatment. [ ] indicate intermittent therapy; subscript number after [ ] indicates number of doses per week.

a n/a, not available, meaning relapse and/or acquired drug resistance (ADR) not measured.

INH, isoniazid resistant; STREP, streptomycin resistant; PDR, poly-drug resistant (streptomycin+isoniazid resistant); FDC, fixed drug combinations.

### Pooled Results across Trials

When results were pooled across all 57 trials, rifampin was given for 1 mo in 12 arms, 2 mo in 60, 5 mo in 6, 6 mo in 170, and 7 mo in 2. Regimens with 1–2 mo of rifampin had higher failure (Table 6) and relapse (Table 7) rates than regimens with longer duration rifampin. Relapse rates were progressively lower with longer rifampin duration, up to 8 mo or more of rifampin (see Figure S1).

**Table 6:** Stratified estimates of treatment failures in RCT in new cases.

Factor	Studies (*N*)	Events/Participants (*N*)	Pooled Event Rate (Across All Trials)	95% CI	I^2^ (95% CI)
**Duration of rifampin**					
Rifampin 1–2 mo	72	94/4,133	1.8	0.2 to 3.3	0.36 (0.15 to 0.52)
Rifampin 3–5 mo	42	16/2,508	0.3	0 to 0.6	0 (0 to 0.35)
Rifampin 6–7 mo	178	150/10,060	0.4	0.1 to 0.7	0 (0 to 0.19)
Rifampin 8+ mo	18	10/1,384	0.2	0 to 0.6	0 (0 to 0.49)
**Use of intermittent therapy**					
Daily throughout	159	179/11,510	0.4	0.2 to 0.7	0.07 (0 to 0.24)
Daily then thrice weekly	35	4/961	0.3	0 to 1.0	0 (0 to 0.38)
Daily then twice weekly	46	49/2,749	1.2	0.1 to 2.4	0.21 (0 to 0.45)
Thrice weekly throughout^a^	70	38/2,865	0.5	0 to 1.0	0 (0 to 0.28)
**Initial drug resistance**					
DST not done/reported	19	78/2,105	2.2	0 to 4.4	0 (0 to 0.48)
Sensitive to all TB drugs	126	120/14,900	0.3	0.1 to 0.4	0 (0 to 0.21)
Isoniazid resistance	67	25/477	**2.8**	**0.7 to 5.0**	0 (0 to 0.29)
Streptomycin resistance	54	6/316	1.3	0 to 2.7	0 (0 to 0.31)
INH+streptomycin resistant (PDR)	44	41/287	**8.3**	**1.9 to 14.7**	0 (0 to 0.34)
**Duration of pyrazinamide**					
No pyrazinamide	59	97/4,831	0.3	0 to 0.6	0.30 (0.03 to 0.49)
1–3 mo	139	124/8,287	0.6	0.2 to 1.0	0 (0 to 0.21)
4+ mo	112	49/4,967	0.5	0.1 to 0.8	0 (0 to 0.23)
**Duration of streptomycin**					
No streptomycin	100	188/7,907	0.6	0.2 to 0.9	0.18 (0 to 0.36)
1–3 mo	117	44/6,328	0.4	0.1 to 0.6	0 (0 to 0.23)
4+ mo	93	38/3,850	0.5	0 to 0.9	0 (0 to 0.25)
**Number of drugs to which strains susceptible** b					
*Initial phase*					
0–1 drugs	2	10/29	33.2	0 to 103.5	0 (–, –)
2 drugs	66	114/1,782	2.8	0.2 to 5.2	0.52 (0.36 to 0.63)
3 drugs	151	43/5,664	0.3	0 to 0.5	0 (0 to 0.20)
4 drugs	72	25/8,505	0.1	0 to 0.1	0 (0 to 0.28)
*Continuation phase*					
0–1 drugs	69	54/588	**2.6**	**0 to 6.1**	0 (0 to 0.28)
2 drugs	142	113/9,838	0.2	0.1 to 0.4	0 (0 to 0.20)
3 or more drugs	74	25/5,528	0.1	0 to 0.2	0 (0 to 0.27)
**Supervision of therapy**					
All doses fully supervised	232	145/10,446	0.4	0.1 to 0.7	0 (0 to 0.16)
None or partial DOT	78	125/7,639	0.4	0.1 to 0.7	0.19 (0 to 0.39)
**Completion of therapy**					
Good (≤10% dropouts)	181	102/11,837	0.3	0.1 to 0.5	0 (0 to 0.19)
Poor (>10% dropouts)	129	168/6,248	0.9	0.3 to 1.5	0.25 (0.07 to 0.40)

Event rate and 95% CI are in bold if confidence intervals for two or more strata do not overlap.

a In all but one trial, if therapy was intermittent initially, the same schedule was continued throughout therapy.

b In a few trials, the number of drugs was the same throughout—these were classified according to the starting regimen.

**Table 7 pmed-1000146-t007:** Stratified estimates of relapse in RCT in new cases.

Factor	Studies (*N*)	Events/Participants (*N*)	Pooled Event Rate (Across All Trials)	95% CI	I^2^ (95% CI)
**Overall**					
*Duration of rifampin*					
Rifampin 1–2 mo	70	367/3,349	**16.0**	**11.1 to 20.9**	0.67 (0.58 to 0.74)
Rifampin 3–5 mo	42	185/2,389	**7.1**	**4.5 to 9.7**	0.65 (0.52 to 0.75)
Rifampin 6–7 mo	171	364/8,639	**3.8**	**2.9 to 4.7**	0 (0 to 0.19)
Rifampin 8+ mo	18	14/1,181	1.0	0.2 to 1.7	0 (0 to 0.46)
*Use of intermittent therapy*					
Daily throughout	153	566/9,829	4.8	3.6 to 6.0	0.56 (0.49 to 0.64)
Daily then thrice weekly	34	33/907	2.9	0.7 to 5.2	0 (0 to 0.38)
Daily then twice weekly	44	181/2,367	7.3	4.0 to 10.7	0.6 (0.45 to 0.71)
Thrice weekly throughout^a^	70	150/2,455	5.7	3.1 to 8.3	0.23 (0 to 0.43)
**Initial drug resistance**					
DST not done/reported	17	124/1,337	7.8	3.0 to 12.5	0.82 (0.73 to 0.88)
Sensitive to all TB drugs	123	684/13,302	3.7	2.8 to 4.7	0.66 (0.59 to 0.72)
Isoniazid resistance	65	60/403	**11.4**	**6.5 to 16.2**	0 (0 to 0.28**)**
Streptomycin resistance	54	36/299	9.7	4.6 to 14.9	0 (0 to 0.32)
INH+streptomycin resistant (PDR)	42	26/217	10.1	4.2 to 15.9	0 to (0 to 0.34)
**Duration of pyrazinamide**					
No pyrazinamide	56	197/3,532	5.1	2.8 to 7.4	0.67 (0.58 to 0.75)
1–3 mo	136	445/7,539	4.9	3.5 to 6.4	0.46 (0.34 to 0.56)
4+ mo	109	288/4,487	6.1	3.9 to 8.2	0.38 (0.22 to 0.51)
**Duration of streptomycin**					
No streptomycin	95	286/6,277	2.7	1.8 to 3.6	0.44 (0.29 to 0.56)
1–3 mo	115	441/5,680	7.5	5.5 to 9.6	0.65 (0.61 to 0.73)
4+ mo	91	203/3,601	5.6	3.4 to 7.7	0.27 (0.06 to 0.44)
**Number of drugs to which strains susceptible** b					
*Initial phase*					
0–1 drugs	2	2/17	9.3	0.0 to 30.2	0 (–, –)
2 drugs	63	72/1,210	6.6	2.7 to 10.4	0.06 (0 to 0.31)
3 drugs	148	284/5,191	4.1	2.6 to 5.6	0.36 (0.22 to 0.47)
4 drugs	71	448/7,803	4.1	2.4 to 5.8	0.66 (0.57 to 0.74)
*Continuation phase*					
0–1 drugs	66	56/487	7.6	3.3 to 11.9	0 (0 to 0.28)
2 drugs	140	438/8,884	3.8	2.5 to 5.1	0.54 (0.45 to 0.62)
3 or more drugs	72	307/4,824	4.5	2.4 to 6.5	0.54 (0.40 to 0.65)
**Supervision of therapy**					
All doses fully supervised	225	693/9,323	7.3	5.8 to 8.8	0.48 (0.39 to 0.55)
None or partial DOT	76	237/6,235	2.4	1.6 to 3.2	0.43 (0.26 to 0.56)
**Completion of therapy**					
Good (≤10% dropouts)	175	640/10,340	5.5	4.1 to 6.8	0.56 (0.48 to 0.63)
Poor (>10% dropouts)	126	290/5,218	4.6	3.2 to 6.1	0.42 (0.29 to 0.53)

Event rate and 95% CI are in bold if confidence intervals for two or more strata do not overlap.

a In all but one trial, if therapy was intermittent initially, the same schedule was continued throughout therapy.

b In a few trials the number of drugs was the same throughout—these were classified according to the starting regimen.

With regard to the different intermittent schedules compared, evidence regarding therapy given twice weekly throughout was limited to a single study of 223 patients who received 12 mo of isoniazid and rifampin—daily or twice weekly [23],[27]. Rates of failure, relapse, and acquired drug resistance were less than 1%. Because this was a single study, these results could not be pooled and are not considered further in stratified or multivariate analyses. The other schedules, of daily, daily then thrice weekly, daily then twice weekly, and thrice weekly throughout, were evaluated in numerous studies. As seen in Tables 6–8, there were no significant differences in outcomes between these four different treatment schedules.

**Table 8:** Stratified estimates of acquired drug resistance in RCT in new cases.

Factor	Arms (*N*)	Events/Participants (*N*)	Pooled Event Rate (Across All Trials)	95% CI	I^2^ (95% CI)
**Overall**					
*Rifampin use* a					
Rifampin 1–2 mo	61	41/2,847	0.8	0 to 1.6	0 (0 to 0.28)
Rifampin 3–5 mo	33	10/1,932	0.3	0 to 0.6	0 (0 to 0.35)
Rifampin 6–7 mo	146	60/7,180	0.4	0.1 to 0.7	0 (0 to 0.19)
Rifampin 8+ mo	17	6/1,249	0.2	0 to 0.5	0 (0 to 0.46)
*Use of intermittent therapy* a					
Daily throughout	125	67/8,541	0.3	0.1 to 0.6	0 (0 to 0.20)
Daily then thrice weekly	28	3/636	0.6	0 to 1.8	0 (0 to 0.38)
Daily then twice weekly	36	12/1,748	0.4	0 to 1.0	0 (0 to 0.34)
Thrice weekly throughout	68	35/2,283	0.9	0 to 2.0	0 (0 to 0.28)
**Initial drug resistance**					
Sensitive to all TB drugs	106	70/12,256	0.3	0.1 to 0.4	0 (0 to 0.22)
Isoniazid resistance	58	13/380	**2.4**	**0.5 to 4.4**	0 (0 to 0.29)
Streptomycin resistance	52	12/313	2.6	0.3 to 5.0	0 (0 to 0.32)
INH+streptomycin resistant (PDR)	41	22/259	**5.7**	**1.3 to 10.1**	0 (0 to 0.34)
**Duration of pyrazinamide**					
No pyrazinamide	48	26/3,662	0.2	0 to 0.3	0 (0 to 0.30)
1–3 mo	113	46/5,536	0.5	0.1 to 0.9	0 (0 to 0.20)
4+ mo	96	45/4,010	0.5	0.1 to 1.0	0 (0 to 0.23)
**Duration of streptomycin**					
No streptomycin	68	31/4,314	0.3	0 to 0.5	0 (0 to 0.24)
1–3 mo	101	51/5,585	0.4	0.1 to 0.7	0 (0 to 0.23)
4+ mo	88	35/3,309	0.8	0.1 to 1.6	0 (0 to 0.25)
**Number of drugs to which strains susceptible** b					
*Initial phase*					
0–1 drugs	1	6/17	34.5	0 to 107.7	0 (–, –)
2 drugs	57	26/919	**2.8**	**0.6 to 5.0**	0 (0 to 0.29)
3 drugs	136	47/4,899	0.5	0.2 to 0.8	0 (0 to 0.20)
4 drugs	63	38/7,373	0.2	0 to 0.3	0 (0 to 0.28)
*Continuation phase*					
0–1 drugs	62	26/511	**2.8**	**0.4 to 5.3**	0 (0 to 028)
2 drugs	126	65/8,037	0.4	0.1 to 0.6	0 (0 to 0.20)
3 or more drugs	63	26/4,634	0.2	0 to 0.4	0 (0 to 0.27)
**Supervision of therapy**					
All doses fully supervised	200	101/8,364	0.7	0.3 to 1.2	0 (0 to 0.17)
None or partial DOT	57	16/4,844	0.1	0 to 0.3	0 (0 to 0.27)
**Completion of therapy**					
Good (≤10% dropouts)	148	71/9,483	0.3	0.1 to 0.5	0 (0 to 0.19)
Poor (>10% dropouts)	109	46/3,725	1.1	0.3 to 1.8	0 (0 to 0.22)

Event rate and 95% CI are in bold if confidence intervals for two or more strata do not overlap.

a In all but one trial, if therapy was intermittent initially, the same schedule was continued throughout therapy.

b In a few trials the number of drugs was the same throughout—these were classified according to the starting regimen.

Of the other factors considered, initial drug resistance was associated with increased risk of failure, relapse, and acquired drug resistance. In the presence of initial isoniazid or isoniazid and streptomycin resistance (poly-drug resistance ), regimens using 1–2 mo of rifampin had failure rates of 6.5% (95% CI: 0.7%–12.3%) or 29% (10%–53%), respectively, in comparison with 0.2% (0%–0.5%) in pan-sensitive TB, and relapse rates of 38% (29%–46%) or 27% (10%–44%), respectively, compared to 8.2% (4.5%–11.9%) in pan-sensitive TB. Acquired drug resistance was also increased if there was initial drug resistance, but this effect appeared similar with all durations of rifampin. Interestingly, failure and acquired drug resistance were progressively lower with use of more drugs to which the organisms were sensitive in the initial phase (up to four drugs), and also associated (but less strongly) with the number of drugs to which the organisms were sensitive in the continuation phase.

### Meta-Regression

When adjusted for potentially confounding treatment factors in multivariate regression, regimens with 1–2 mo of rifampin were associated with significantly higher failure, relapse, and acquired drug resistance rates than the reference group of 6 mo of rifampin (Table 9). Interestingly, adjusted relapse rates were lower with regimens using rifampin for at least 8 mo than with the 6-mo rifampin reference group. None of the intermittent schedules was significantly associated with failure or relapse, although acquired drug resistance was increased with two of the three intermittent regimens. Streptomycin use was protective against failure, relapse, and acquired drug resistance, while pyrazinamide use was protective only for relapse.

**Table 9:** Adjusted incidence rate ratios of failure, relapse, and acquired drug resistance (from negative binomial regression).

Factor	Failure IRR (95% CI)	Relapse IRR (95% CI)	Acquired Drug Resistance^a^ IRR (95% CI)
**Duration of rifampin** b			
1–2 mo	**5.8 (2.9 to 11.0)**	**3.6 (2.5 to 5.3)**	**4.6 (2.0 to 0.4)**
3–4 mo	1.3 (0.6 to 3.0)	**2.6 (1.6 to 4.0)**	1.2 (0.4 to 3.1)
5–7 mo	1.0 (reference)	1.0 (reference)	1.0 (reference)
8+ mo	2.0 (0.8 to 4.9)	**0.4 (0.2 to 0.7)**	2.1 (0.8 to 5.3)
Overall significance (*p* value)^c^	(<0.0001)	(<0.0001)	(<0.002)
**Schedule of drug administration** b			
Daily throughout	1.0 (reference)	1.0 (reference)	1.0 (reference)
Daily then thrice weekly	0.7 (0.2 to 2.1)	1.0 (0.6 to 1.5)	0.7 (0.2 to 2.6)
Daily then twice weekly	0.9 (0.5 to 1.6)	0.8 (0.5 to 1.2)	0.5 (0.3 to 1.2)
Thrice weekly throughout	0.7 (0.3 to 1.4)	1.2 (0.8 to 1.6)	**2.4 (1.05 to 5.5)**
Overall significance (*p* value)^c^	(0.66)	(0.38)	(0.02)
**Other Factors**			
*Initial drug resistance* d			
DST not done/reported	**3.3 (1.5 to 7.2)**	**3.0 (1.6 to 4.9)**	N/A
Pan-sensitive strain	1.0 (reference)	1.0 (reference)	1.0 (reference)
Isoniazid resistant	**10.9 (5.9 to 20)**	**1.8 (1.2 to 2.6)**	**5.1 (2.3 to 11.0)**
Streptomycin resistant	**3.9 (1.4 to 11.0)**	1.4 (0.9 to 2.2)	**4.1 (1.6 to 10.0)**
Poly-drug resistant (PDR)	**33 (16 to 62)**	**1.8 (1.1 to 2.9)**	**10.0 (4.5 to 22.1)**
Overall significance (*p* value)^c^	(<0.0001)	(<0.0001)	(<0.0001)
**Use of pyrazinamide** b			
Pyrazinamide not used	1.0 (reference)	1.0 (reference)	1.0 (reference)
Pyrazinamide used	**4.7 (2.4 to 9.0)**	**0.7 (0.4 to 0.95)**	**2.4 (1.1 to 4.9)**
Overall significance (*p* value)^c^	(<0.0001)	(0.04)	(0.02)
**Use of streptomycin** b			
Streptomycin not used	1.0 (reference)	1.0 (reference)	1.0 (reference)
Streptomycin used	**0.3 (0.2 to 0.6)**	0.9 (0.6 to 1.3)	0.7 (0.4 to 1.3)
Overall significance (*p* value)^c^	(0.0003)	(0.67)	(0.3)
Number of drugs to which strains susceptible*			
*Initial phase*			
0–1 drugs	**99 (33 to 99)**	1.6 (0.2 to 11.0)	**74 (8.1 to 99)**
2 drugs	**20 (8.2 to 49)**	1.1 (0.6 to 1.8)	**6.7 (2.8 to 16)**
3 drugs	**2.6 (1.3 to 5.0)**	1.1 (0.8 to 1.5)	**2.9 (1.5 to 5.5)**
4 or more drugs	1.0 (reference)	1.0 (reference)	1.0 (reference)
Overall significance (*p* value)^c^	(<0.0001)	(0.9)	(0.0004)
*Continuation phase*			
0–1 drugs	1.1 (0.4 to 2.6)	1.2 (0.7 to 2.0)	**2.9 (1.1 to 7.3)**
2 drugs	**0.5 (0.2 to 0.9)**	0.8 (0.5 to 1.05)	1.7 (0.9 to 3.2)
3 or more drugs	1.0 (reference)	1.0 (reference)	1.0 (reference)
Overall significance (*p* value)^c^	(0.01)	(0.08)	(0.08)

a Acquired drug resistance in both failure and relapse cases combined.

b Adjusted estimates of Incidence Rate Ratios (IRR) from multivariate negative binomial regression with model that included all variables indicated, plus length of follow-up after end of treatment (for relapse and acquired drug resistance), supervision of therapy (DOT), and non-completion of therapy because of protocol violations, patient refusal, default, moved, or lost. Estimates that are statistically significant are in bold.

c Overall significance of each factor in multi-variate models, from log likelihood ratio test.

d Adjusted estimates of IRR from second model that included initial drug resistance, and all the same factors, but not the number of sensitive drugs in initial or continuation phases. These could not be included because of substantial co-linearity with drug resistance.

The proportion smear positive, proportion HIV infected, and duration of post-treatment follow-up were not associated with treatment outcomes and were not included in final regression models. Poor completion of treatment was associated with failure or relapse, whereas supervision of therapy was not; both factors were included in final multivariate models, although estimates are not shown. Dispersion estimates for all three final models were less than 1, suggesting that the treatment factors included in these models accounted for the majority of the heterogeneity in outcomes seen.

## Discussion

In this review of 57 trials with rifampin-containing regimens, use of rifampin only initially rather than throughout treatment was associated with worse treatment outcomes (higher rates of failure, relapse, and acquired drug resistance). Thrice-weekly intermittent dosing schedules during the initial treatment phase were associated with increased adjusted risk of acquired drug resistance, but not relapse or failure. Initial drug resistance was strongly associated with increased risk of poor treatment outcomes, particularly if rifampin was used only in the initial intensive phase. These findings have important implications for TB treatment.

The most important finding of this review is that all three treatment outcomes were significantly worse with regimens that used rifampin for the first 1–2 mo rather than throughout therapy. This finding adds considerable weight to similar findings by Jindani and colleagues, who compared regimens containing 2 mo versus 6 mo of rifampin [26]. This review includes many more studies with a variety of regimens, making these results more robust and generalizable. In this review, the failure and relapse rates progressively declined with long duration of rifampin; such a dose–response relationship strengthens the conclusions that a longer duration of rifampin treatment is responsible for better outcomes. Finally, this review included studies where drug sensitivity testing was performed, which permitted us to detect an increased risk of acquired drug resistance with shorter rifampin duration and also permitted stratified analysis by underlying drug resistance, which proved to be a very important determinant of treatment outcomes. According to the most recent information from WHO [96], the 8-mo regimen was the recommended initial therapy in 24 high-incidence countries. Based on the pooled risk differences from within trial analyses, we estimate that treatment of 100 patients with the regimen of 2HRZE/6HE (the “8-mo” regimen) would result in 13 more failures and relapses than if they received 2HRZE/4HR (the “6-mo” regimen). As a result, forthcoming recommendations by WHO will recommend only the 6-mo (rifampin throughout) regimen, and the 8-mo regimen will no longer be recommended [97]. Results of this review suggest that the public health benefits of switching from the 8-mo to the 6-mo regimen should be very considerable.

The lower risk of relapse with regimens using rifampin for at least 8 mo is consistent with subgroup analyses of other trials [98] and a recent cohort report from Hong Kong [99]. These have shown that patients with extensive cavitary pulmonary disease have increased risk of relapse with 6-mo regimens. Taken together these findings support recommendations to extend therapy for patients at high risk of relapse [13]. However, accurate identification of high-risk patients is imprecise, and provision of extended therapy may be challenging in high-burden settings.

The lack of effect of intermittency is interesting but has several caveats. The timing of intermittent dosing may be quite important, as suggested by the finding of increased risk of acquired resistance associated with thrice-weekly therapy throughout. A cohort study from New York City reported that patients with HIV–TB coinfection had an increased risk of acquired rifamycin resistance if they were treated with twice-weekly therapy during the initial intensive phase but not if they were treated with intermittent dosing only during the continuation phase [100].

Te other important finding is the previously underestimated impact of primary isoniazid resistance on failure, relapse, and acquired resistance. This important effect is a powerful argument for widespread availability of rapid, inexpensive testing for resistance to isoniazid (as well as for rifampin), or for regimens that do not require optimal activity from isoniazid. The influence of primary streptomycin resistance is likely to be less important, since streptomycin has been replaced by ethambutol in most settings.

The primary objective of this review was to compare the efficacy of different durations and dosing schedule of rifampin. To accomplish this, we have analyzed the per-protocol results from each trial, using standardized microbiological definitions. All studies reviewed reported adverse events, dropouts, and defaulters separately, facilitating our approach. However, we did not include these outcomes because they are not as well defined nor standardized, potentially creating greater between-study variability. As well, inter-study differences in providers and populations could have very important influences on these outcomes—even greater than any biologic differences in disease response. These would be balanced within each trial but could have created substantial bias with our analytic approach. If dropout, default, or side effects were associated with the same characteristics as failure or relapse, then excluding these outcomes could underestimate the poor outcomes associated with shorter rifampin exposures. But if not, then including these outcomes would simply reduce all differences between regimens.

This review had several limitations. First, we could identify few trials with direct head-to-head comparisons of rifampin duration, and even fewer directly comparing intermittent regimens. Hence, we had to pool results across studies; this increases potential confounding from differences in treatment, patients' disease severity, or other differences in the study populations, since the studies were conducted in many different countries. The advantage of this approach is that we are able to include many more trials, thereby increasing the precision and avoiding selection bias [101]. However, the disadvantage is the greater potential for bias due to between-trial differences in participant characteristics, treatment regimens, as well as the differential impact of dropouts and other losses to follow-up [102]. Concern about this latter problem should be alleviated by the consistent results from three analytic approaches—within the smaller set of trials with head-to-head comparisons, across all 57 trials, and the multivariate analysis. Also, the dispersion estimates from multivariable analysis suggest that treatment factors and underlying drug resistance accounted for almost all the differences in outcomes observed.

Most trials were initiated before 1980, limiting the number of participants with HIV infection and drug resistance. The lack of trials in HIV infected persons with active TB meant that the question of rifampin duration in treatment of HIV-TB could not be answered, due to insufficient power. This underscores the paucity of recent TB treatment trials and the urgent need for trials in drug resistant and/or HIV infected populations. There were no trials in children, reflecting a lack of rigorous trials in this population and the difficulties of microbiologic confirmation in this population. Death was not analyzed, because most TB-related deaths occur soon after diagnosis and are determined by comorbidity, age, severity of illness, and delay in diagnosis [103],[104]. Deaths later in treatment are often from other causes [105],[106]. Hence, differences in the TB treatment regimen may have relatively little impact on mortality. We endeavored to minimize language bias by including studies published in French and Spanish as well as English. Interestingly, this yielded only three additional trials, or 5% of all trials included. In a recent review [107], of all TB related papers listed in PubMed over 10 y, papers published in English, French, and Spanish represented 84% of all published literature worldwide. Hence, this review can be considered reasonably representative of publications in this field. However, in some fields, such as mental health, PubMed will fail to list a substantial proportion of relevant publications from low- to middle-income countries [108], so we may have missed some important trials.

Finally, we were not able to distinguish between relapse of the same strain of *M. tuberculosis* that caused the initial infection and reinfection with a new strain of the bacillus. In settings with high rates of ongoing exposure to *M. tuberculosis*, particularly if HIV seroprevalence is also high, a relatively high proportion of cases of recurrent TB following initial apparent cure may be due to reinfection [109]. However, very few participants had HIV coinfection in the studies reviewed, and in studies with longer follow-up, the great majority of relapses occurred in the first 1–2 y, with very few occurring in the third to fifth years. This suggests that reinfection should have accounted for very few of the disease recurrences. Because follow-up was adequate in almost all studies—only four studies had less than 1-y follow-up—unequal follow-up should not have affected results—supported by the finding that duration of post-treatment follow-up was not associated with relapse rates in multivariable analysis.

### Conclusion

This review provides evidence against continued use of regimens that utilize rifampin for the first 2 mo only, as they are significantly and substantially inferior to regimens that use rifampin for at least 6 mo. This review also has identified an important need for adequately powered clinical trials that address dosing schedules, management of isoniazid mono-resistance, and the optimal duration of treatment to prevent relapse.

## Supporting Information

Figure S1 Forest plots of relapse rates with different duration of rifampin. Only patients with drug-sensitive organisms in studies where drug-sensitive testing was performed are shown.
(3.53 MB TIF)

Text S1 Characteristics of studies reviewed. Appendix Table 1: Rifampin duration directly compared, and regimens otherwise comparable; Appendix Table 2: Intermittent regimens, directly compared head to head within studies and with otherwise comparable regimens; Appendix Table 3: Regimens differed by rifampin duration but also by other important factors; Appendix Table 4: Regimens differed by intermittent schedule, but also by other important factors; Appendix Table 5: No internal comparison of rifampin duration, nor of intermittent schedules; Appendix Table 6: Randomized trials in which only one arm could be analyzed.
(0.40 MB DOC)

## Abbreviations

DOT: directly observed therapy
MDR: multi-drug resistance
TB: tuberculosis
WHO: World Health Organization
