# Supplementary figures and images for: Effect of Duration and Intermittency of Rifampin on Tuberculosis Treatment Outcomes: A Systematic Review and Meta-Analysis
Source: PLoS Med. 2009 Sep 15;6(9):e1000146. doi: 10.1371/journal.pmed.1000146 (PMC2736385; doi:10.1371/journal.pmed.1000146)

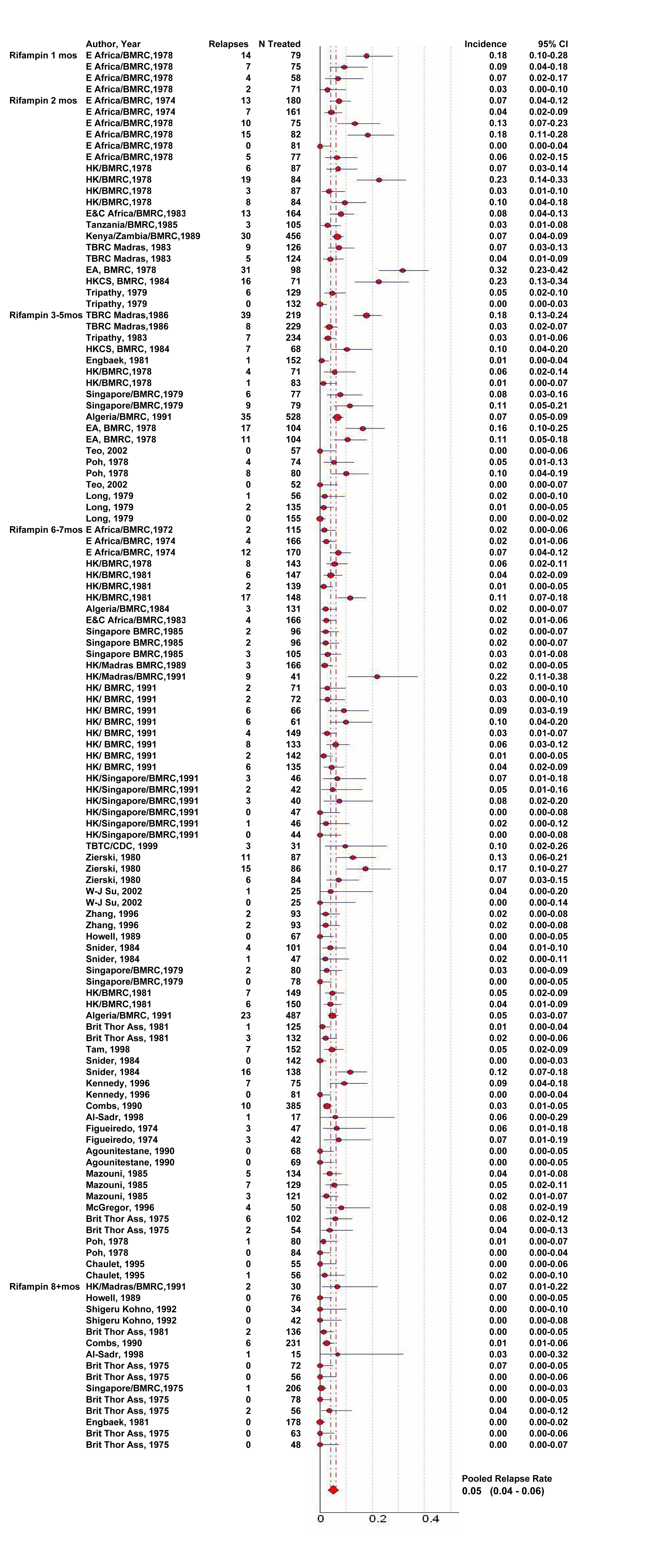

Supplement: Figure S1 — Forest plots of relapse rates with different duration of rifampin. Only patients with drug-sensitive organisms in studies where drug-sensitive testing was performed are shown. (3.53 MB TIF) [file pmed.1000146.s001.tif]
