# Supplementary material for: Effect of Duration and Intermittency of Rifampin on Tuberculosis Treatment Outcomes: A Systematic Review and Meta-Analysis
Source: PLoS Med. 2009 Sep 15;6(9):e1000146. doi: 10.1371/journal.pmed.1000146 (PMC2736385; doi:10.1371/journal.pmed.1000146)
Supplement: Text S1 — Characteristics of studies reviewed. Appendix Table 1: Rifampin duration directly compared, and regimens otherwise comparable; Appendix Table 2: Intermittent regimens, directly compared head to head within studies and with otherwise comparable regimens; Appendix Table 3: Regimens differed by rifampin duration but also by other important factors; Appendix Table 4: Regimens differed by intermittent schedule, but also by other important factors; Appendix Table 5: No internal comparison of rifampin duration, nor of intermittent schedules; Appendix Table 6: Randomized trials in which only one arm could be analyzed. (0.40 MB DOC) [file pmed.1000146.s002.doc]

**APPENDIX TABLE 1. Characteristics of Studies Reviewed – By Type of Comparisons Made.**

**RIFAMPIN duration directly compared, and regimens otherwise comparable.**

|  | **Author,**  **Year**  **(references)** | **Country** | **Drug Resistance**  **Patterns** | **Treatment Regimens** | **Number at risk for Failure** | **Number at risk for Relapse** |
| --- | --- | --- | --- | --- | --- | --- |
| **2 months vs. 3 or 4 months** | | | | | | |
|  | EA, BMRC, 1978 [1,2] | International | Pan-sensitive, STREP,  INH, PDR | 2SHRZ/2HR | 122 | 112 |
|  |  |  |  | 2SHRZ/2HRZ | 127 | 118 |
|  |  |  |  | 2SHRZ/2HZ | 124 | 116 |
|  | HKCS, BMRC, 1984 [3,4] | Hong Kong | Pan-sensitive | 2SHRZ | 84 | 84 |
|  |  |  |  | 3SHRZ | 81 | 81 |
| **2 months vs. 6 months** | | | | | | |
|  | BMRC, 1983  [5,6] | East and Central Africa | Pan-sensitive, STREP,  INH, PDR | 2SHRZ/4HR | 212 | 177 |
|  |  |  |  | 2SHRZ/4HZ | 209 | 181 |
|  | Jindani, 2004[7] | International | Pan-sensitive plus all forms of resistance except MDR | 2EHRZ/4HR | 383 | 242 |
|  |  |  |  | 2EHRZ/6HE | 402 | 203 |
|  |  |  |  | 2[EHRZ]_3_/6HE | 410 | 198 |
|  | Felten, 1989 [8] | South Africa | Pan-sensitive plus all forms of resistance except MDR | 2HRZE/4[HRZ]_2_ | 54 | 51 |
|  |  |  |  | 2HRZE/4[HZE]_2_ | 44 | 41 |
| **4 months vs. 6 months** | | | | | | |
|  | BMRC, 1979 [9–11] | Singapore | Pan-sensitive, STREP,  INH, PDR | 2SHRZ/2HR | 91 | 87 |
|  |  |  |  | 2SHRZ/4HR | 90 | 85 |
|  |  |  |  | 2SHRZ/2HRZ | 91 | 89 |
|  |  |  |  | 2SHRZ/4HRZ | 89 | 82 |
|  | Sharifi-Mood, 2006 [12] | Iran | Not done | 2HRZE/2HR | 33 | 33 |
|  |  |  |  | 2HRZE/4HR | 67 | 64 |
|  | Poh, 1978 [13] | Singapore | Pan-sensitive | 2SHRZ/2HR | 79 | 74 |
|  |  |  |  | 2SHRZ/4HR | 83 | 80 |
|  |  |  |  | 2SHRZ/2HRZ | 83 | 80 |
|  |  |  |  | 2SHRZ/4HRZ | 87 | 84 |
| **6 months vs. 9 months** | | | | | | |
|  | BMRC, 1991 [14] | Hong Kong | Pan-sensitive, STREP,  INH, PDR | 6SHRZ.3 | 68 | 56 |
|  |  |  |  | 8SHRZ.3 | 54 | 42 |
|  | Brit Thor Ass, 1975 [15–17] | UK | Pan-sensitive plus all forms of resistance except MDR | 2EHR/10HR | 104 | 78 |
|  |  |  |  | 2EHR/16HR | 93 | 63 |
|  |  |  |  | 2EHR/4HR | 114 | 102 |
|  |  |  |  | 2EHR/7HR | 85 | 72 |
|  |  |  |  | 2SHR/10HR | 79 | 56 |
|  |  |  |  | 2SHR/16HR | 62 | 48 |
|  |  |  |  | 2SHR/4HR | 74 | 54 |
|  |  |  |  | 2SHR/7HR | 72 | 56 |
|  | Perriens, 1995 [18] | Zaire | Pan-sensitive plus all forms of resistance except MDR | 2HRZE/10[HR]_2_ | 135 | 54 |
|  |  |  |  | 2HRZE/4[HR]_2_ | 134 | 68 |
|  | Combs, 1990 [19] | USA | Pan-sensitive | 2HRZ/4HR |  | 385 |
|  |  |  |  | 2HRZ/7HR |  | 231 |
|  | El-Sadr, 1998 [20]* | USA | Pan-sensitive | 2HRZE/4HR | 42 | 17 |
|  |  |  |  | 2HRZE/7HR | 37 | 15 |

| **OTHER COMPARISONS** | | | | | | |
| --- | --- | --- | --- | --- | --- | --- |
| **3 months vs. 18 months** | | | | | | |
|  | Engbaek, 1981 [21] | Denmark | Pan-sensitive | 3HRE/15HE | 191 | 152 |
|  |  |  |  | 3HRE/15HR | 211 | 178 |
| **6 months plus PZA vs. 9 months without PZA** | | | | | | |
|  | Howell, 1989 [22] | Ireland | Pan-sensitive | 2RHE/7RH | 76 | 76 |
|  |  |  |  | 2RHEZ/4RH | 67 | 67 |
|  | Brit Thor Ass, 1980 [23,24] | UK | Pan-sensitive | 2EHR/7HR | 157 | 136 |
|  |  |  |  | 3EHRZ/4HR | 151 | 132 |
|  |  |  |  | 2SHRZ/4HR | 146 | 125 |

**APPENDIX TABLE 2. Characteristics of Studies Reviewed – By Type of Comparisons Made.**

**Intermittent regimens – directly compared head to head within studies, and with otherwise comparable regimens.**

|  | **Author, Year**  **(references)** | **Country** | **Drug Resistance Patterns** | **Drug regimens †** | **Number at risk for Failure** | **Number followed for Relapse** |
| --- | --- | --- | --- | --- | --- | --- |
|  | Zierski, 1980 [25] | Poland | Pan-sensitive, INH, SDR | 2HRE/4[HR]_2_ | 93 | 89 |
|  |  |  |  | 2HRE/4[HRE]_2_ | 96 | 89 |
|  |  |  |  | 6HRE | 98 | 92 |
|  | HKCS BMRC, 1987 [26,27] | Hong Kong | Pan-sensitive, STREP, INH, PDR | 6HRZE | 199 | 180 |
|  |  |  |  | 6[HRZE]_3_ | 199 | 171 |
|  | Teo, 1990 [28] | Singapore | Pan-sensitive | 2HRZ/2HR | 59 | 57 |
|  |  |  |  | 2HRZ/4[HR]_3_ | 54 | 52 |
|  | MacNab, 1990 [29] | South Africa | Pan-sensitive plus all forms of resistance except MDR | 3[HRZE]_5_/3.5[HR]_5_ | 39 |  |
|  |  |  |  | 6.5HRZ | 67 |  |
|  | Jindani, 2004 [7] | International | Pan-sensitive plus all forms of resistance except MDR | 2EHRZ/6HE | 402 | 203 |
|  |  |  |  | 2[EHRZ]_3_/6HE | 410 | 198 |
| **Notes:** STREP = Streptomycin resistant, INH=Isoniazid resistance, PDR**=** poly-drug resistance **(**Streptomycin & Isoniazid resistant)  * In the El-Sadr study, patients were initially randomized to 5drugs vs. 4drugs in the initial intensive phase. Then they were re-randomized to longer or shorter total duration of therapy. The addition of the fifth drug in the initial intensive phase was ignored in this analysis as this has no effect on outcomes.  **†** Regimen abbreviations: H=Isoniazid; R=Rifampin; Z=Pyrazinmade; E=Ethambutol; S=Streptomycin  Letters to left of ‘/’ indicate regimen in initial intensive phase, and letters to right of ‘/’ indicate regimen in continuation phase. First number equals months of initial phase of treatment and the second number equals months of continuation phase treatment.  [ ] indicate intermittent therapy, and subscript number after [ ] indicate number of doses per week. | | | | | | |

**APPENDIX TABLE 3. Characteristics of Studies Reviewed – By Type of Comparisons Made.**

**Regimens differed by Rifampin Duration but also by other important factors.**

|  | **Author, Year**  **(references)** | **Country** | **Drug Resistance Patterns** | **Treatment Regimens** | **Number at risk for Failure** | **Number followed for Relapse** |
| --- | --- | --- | --- | --- | --- | --- |
|  | BMRC, 1974 [30,31] | East Africa | Pan-sensitive | 2SHRZ/4[SHZ]_2_ | 179 | 161 |
|  |  |  |  | 2SHRZ/4TH | 191 | 180 |
|  |  |  |  | 6HR | 183 | 170 |
|  |  |  |  | 6SHR | 181 | 166 |
|  | BMRC, 1978 [32,33] | Hong Kong | Pan-sensitive, STREP,  INH, PDR | 2SHRE/4[SHE]_2_ | 111 | 105 |
|  |  |  |  | 2SHRE/6[SHE]_2_ | 112 | 108 |
|  |  |  |  | 2SHRZ/4[SHZ]_2_ | 112 | 101 |
|  |  |  |  | 2SHRZ/6[SHZ]_2_ | 106 | 101 |
|  |  |  |  | 4[SHRZ]_3_/2[SHZ]_2_ | 98 | 86 |
|  |  |  |  | 4[SHRZ]_3_/4[SHZ]_2_ | 108 | 96 |
|  |  |  |  | 6SHR | 195 | 172 |

**APPENDIX TABLE 4. Characteristics of Studies Reviewed – By Type of Comparisons Made.**

**Regimens differed by intermittent schedule, but also by other important factors**

|  | **Author, Year**  **(references)** | **Country** | **Drug Resistance Patterns** | **Treatment Regimens †** | **Number at risk for Failure** | **Number followed for Relapse** |
| --- | --- | --- | --- | --- | --- | --- |
|  | EA BMRC, 1978 [34,35] | E Africa | Pan-sensitive, STREP, INH, PDR | 1SHRZ/5[SHZ]_2_ | 98 | 79 |
|  |  |  |  | 1SHRZ/5TH | 97 | 86 |
|  |  |  |  | 1SHRZ/7[SHZ]_2_ | 111 | 77 |
|  |  |  |  | 1SHRZ/7TH | 82 | 61 |
|  |  |  |  | 2SHR/4TH | 111 | 93 |
|  |  |  |  | 2SHR/6TH | 105 | 83 |
|  |  |  |  | 2SHRZ/4TH | 100 | 82 |
|  |  |  |  | 2SHRZ/6TH | 109 | 91 |
|  | TBRC Madras, 1985 [36,37] | India | Pan-sensitive, STREP, INH, PDR | 3HRZS | 269 | 257 |
|  |  |  |  | 3HRZS/2[HZS]_2_ | 273 | 269 |
| **Notes:** STREP = Streptomycin resistant, INH=Isoniazid resistance, PDR**=** poly-drug resistance **(**Streptomycin & Isoniazid resistant)  **†** Regimen abbreviations: H=Isoniazid; R=Rifampin; Z=Pyrazinmade; E=Ethambutol; S=Streptomycin  Letters to left of ‘/’ indicate regimen in initial intensive phase, and letters to right of ‘/’ indicate regimen in continuation phase. First number equals months of initial phase of treatment and the second number equals months of continuation phase treatment.  [ ] indicate intermittent therapy, and subscript number after [ ] indicate number of doses per week. | | | | | | |

**APPENDIX TABLE 5. Characteristics of Studies Reviewed – By Type of Comparisons Made.**

**No Internal comparison of Rifampin duration, nor of Intermittent schedules.**

| **Author, Year**  **(references)** | **Country** | **Drug Resistance Patterns** | **Treatment Regimens †** | **Number at risk for Failure** | **Number followed for Relapse** |
| --- | --- | --- | --- | --- | --- |
| Long, 1970 [38] | USA | Pan-sensitive | 5HR(450)/12HE | 73 | 56 |
|  |  |  | 5HR(600)/12HE | 153 | 135 |
|  |  |  | 5HR(750)/12HE | 176 | 155 |
| Corpe, 1972 [39] | USA | Pan-sensitive | 5RH | 209 |  |
|  |  |  | 5RHE | 210 |  |
| Snider, 1975 [40] | USA | Pan-sensitive | 6HR | 211 | 138 |
|  |  |  | 6HR/9HE | 216 | 142 |
| Tripathy, 1979 [41] | India | Pan-sensitive, STREP, INH, PDR | 2SHRZ/3[SHZ]_2_ | 129 | 129 |
|  |  |  | 2SHRZ/4[SHZ]_2_ | 45 |  |
|  |  |  | 2SHRZ/5[SHZ]_2_ | 132 |  |
| TBRC Madras, 1983 [42,43] | India | Pan-sensitive | 2SHRZ/3[SHZ]_2_ | 129 | 126 |
|  |  |  | 2SHRZ/5[SHZ]_2_ | 132 | 124 |
| Snider, 1984 [44] | Poland | Pan-sensitive | 2HRZ/4[HR]_2_ | 112 | 101 |
|  |  |  | 2HRZS/4[HR]_2_ | 53 | 47 |
| Singapore BMRC, 1985 [45,46] | Singapore | Pan-sensitive, STREP, INH, PDR | 1SHRZ/5[HR]_3_ | 114 | 107 |
|  |  |  | 2HRZ/4[HR]_3_ | 124 | 116 |
|  |  |  | 2SHRZ/4[HR]_3_ | 112 | 104 |
| Mazouni, 1985 [47] | Algeria | Pan-sensitive, INH | 3HRE/3HR | 212 | 133 |
|  |  |  | 3HRZ/3HR | 205 | 131 |
|  |  |  | 6HR | 215 | 142 |
| Kohno, 1986 [48] | Japan | Pan-sensitive | 9HRE | 62 | 42 |
|  |  |  | 9HRO | 62 | 34 |
| Teo, 1983 [49] | Singapore | Pan-sensitive plus all forms of resistance except MDR | 1SHRZ/5[HR]_3_ | 46 | 43 |
|  |  |  | 1SHRZ/5[HR]_3_ FDC | 41 | 40 |
|  |  |  | 2HRZ/4[HR]_3_ | 47 | 44 |
|  |  |  | 2HRZ/4[HR]_3_ FDC | 43 | 38 |
|  |  |  | 2SHRZ/4[HR]_3_ | 46 | 42 |
|  |  |  | 2SHRZ/4[HR]_3_ FDC | 48 | 40 |
| HKCS BMRC, 1987 [26,27,50] | Hong Kong | Pan-sensitive, STREP,  INH, PDR | 6[HRSE]_3_ | 198 | 175 |
|  |  |  | 6[HRSZ]_3_ | 183 | 159 |
|  |  |  | 6[HRSZE]_3_ | 192 | 178 |
| Kennedy, 1990 [51] | Tanzania | Pan-sensitive | 2HRZE/2HRZ/2HR | 86 | 81 |
|  |  |  | 4HRCX/2HR | 82 | 75 |
| BMRC, 1991  [52] | Algeria | Pan-sensitive, STREP,  INH, PDR | 2SHRZ/4HR | 519 | 518 |
|  |  |  | 2SHRZ/2HR/4H | 568 | 564 |
| HK BMRC, 1991 [53] | Hong Kong | Pan-sensitive, INH, STREP, PDR | 2[HSZR]_3_/2[SHR]_3_/2HR | 173 | 171 |
|  |  |  | 2[HSZR]_3_/2[SHR]_3_/2HR | 81 | 80 |
|  |  |  | 4[HRSZ]_3_/2[HRZ]_3_ | 169 | 166 |
|  |  |  | 4[HRSZ]_3_/2[HRZ]_3_ FDC | 80 | 75 |
|  |  |  | 4[HSZR]_3_/2[HR]_3_ | 157 | 154 |
|  |  |  | 4[HSZR]_3_/2[HR]_3_  FDC | 83 | 81 |
|  |  |  | 6[HRZ]_3_ | 165 | 154 |
|  |  |  | 6[HRZ]_3_ FDC | 74 | 71 |
| HK Singapore BMRC,1991 [54] | Singapore | Pan-sensitive, STREP, INH, PDR | 1SHRZ/5[HR]_3_ | 90 | 89 |
|  |  |  | 1SHRZ/5[HR]_3_ FDC | 6 | 6 |
|  |  |  | 2HRZ/4[HR]_3_ | 91 | 93 |
|  |  |  | 2HRZ/4[HR]_3_  FDC | 3 | 3 |
|  |  |  | 2SHRZ/4[HR]_3_ | 95 | 89 |
|  |  |  | 2SHRZ/4[HR]_3_  FDC | 1 | 1 |
| Chaulet, 1995 [55] | Algeria | Pan-sensitive, INH | 2HRZ/5HR | 80 | 55 |
|  |  |  | 2HRZ/5HR FDC | 88 | 59 |
| Su, 2002 [56] | China | Pan-sensitive | 2HRZE/4HRE | 25 | 25 |
|  |  |  | 2RE/4RE | 26 | 25 |
| Pedral-Sampaio, 2003 [57] | Brazil | Pan-sensitive plus all forms of resistance except MDR | 2HRZ/4HR | 15 |  |
|  |  |  | 2HRZ/4HR (+RhuGm) | 16 |  |
| **Notes:** STREP = Streptomycin resistant, INH=Isoniazid resistance, PDR**=** poly-drug resistance **(**Streptomycin & Isoniazid resistant)  **†** Regimen abbreviations: H=Isoniazid; R=Rifampin; Z=Pyrazinmade; E=Ethambutol; S=Streptomycin  Letters to left of ‘/’ indicate regimen in initial intensive phase, and letters to right of ‘/’ indicate regimen in continuation phase. First number equals months of initial phase of treatment and the second number equals months of continuation phase treatment.  [ ] indicate intermittent therapy, and subscript number after [ ] indicate number of doses per week. | | | | | |

**APPENDIX TABLE 6. Randomized Trials in which only 1 arm could be analysed**

**(The other arm(s) did not have rifampin, or used regimen that was once weekly, or involved mono-therapy).**

| **Author, Year**  **(references)** | **Country** | **Drug Resistance Patterns **** | **Treatment Regimens †** | **Number at risk for Failure** | **Number followed for Relapse** |
| --- | --- | --- | --- | --- | --- |
| E Africa, 1972 [58–60] | E Africa | Pan-sensitive | 6SHR | 99 | 115 |
| BMRC, 1973 [61] | Hong Kong | Pan-sensitive | 3SHR/9HR | 95 |  |
| Figueiredo, 1974 [62] | Brazil | Pan-sensitive , INH | 6HRE | 124 | 102 |
| Singapore BMRC, 1975 [63,64] | Singapore | Pan-sensitive, INH | .5SHR/12[HR]_2_ | 223 | 211 |
| Shennan, 1981 [65] | Swaziland | Not done | 2SHRZ/10HT | 65 |  |
| Tripathy, 1983 [66] | India | Pan-sensitive | 3SHRZ/2[SHZ]_2_ | 235 | 234 |
| Algeria BMRC, 1984 [67] | Algeria | Pan-sensitive, INH, PDR | 2HRZE/4HR | 154 | 146 |
| Tanzania BMRC, 1985 [68] | Tanzania | Pan-sensitive , STREP, INH, PDR | 2SHRZ/4TH | 126 | 115 |
| HK Madras BMRC 1989 [69] | Hong Kong | Pan-sensitive , STREP, INH, PDR | 6[SHRZ]_3_ | 201 | 190 |
| Kenya, Zambia, BMRC, 1989 [70] | E Africa | Pan-sensitive , INH | 2SHRZ/4TH | 543 | 492 |
| Tam, 1991 [71,72] | China | Pan-sensitive , MDR | 2[SHRZ]_3_/4[HR]_3_ | 172 | 152 |
| Agounitestane, 1990 [73] | Algeria | Pan-sensitive , INH | 2HRZ/4HR | 144 | 142 |
| Mohanty, 1993  [74] | India | Pan-sensitive plus all forms of resistance except MDR | 2SHRZ/4HR | 17 | 17 |
| Zhang, 1996 [75] | China | Pan-sensitive | 2HRZ/4HR | 189 | 186 |
| Gonzalez, 1994[76] | Brazil, Thailand, Argentina | Pan-sensitive plus all forms of resistance except MDR | 2HRZE/4HR | 124 | 119 |
| McGregor, 1996 [77] | South Africa | Pan-sensitive | 2RHZE/4[HRE]_2_ | 93 | 50 |

** STREP = Streptomycin resistant, INH=Isoniazid resistance, PDR**=** poly-drug resistance **(**Streptomycin & Isoniazid resistant)

**†** Regimen abbreviations: H=Isoniazid; R=Rifampin; Z=Pyrazinmade; E=Ethambutol; S=Streptomycin

Letters to left of ‘/’ indicate regimen in initial intensive phase, and letters to right of ‘/’ indicate regimen in continuation phase. First number equals months of initial phase of treatment and the second number equals months of continuation phase treatment.

[ ] indicate intermittent therapy, and subscript number after [ ] indicate number of doses per week.

Reference List

1. East African British Medical Research Councils (1981) Controlled clinical trial of five short-course (4 month) chemotherapy regimens in pulmonary tuberculosis. Am Rev Respir Dis 123: 165-170.

2. East African and British Medical Research Councils (1978) Controlled clinical trial of five short-course (4-month) chemotherapy regimens in pulmonary tuberculosis. Lancet 334.

3. Hong Kong Chest Service, Tuberculosis Research Centre M, British Medical Research Council (1984) A controlled trial of 2-month, 3-month, and 12-month regimens of chemotherapy for sputum-smear-negative pulmonary tuberculosis. Am Rev Respir Dis 130: 23-28.

4. Hong Kong Chest Service, Tuberculosis Research Centre M, British Medical Research Council (1981) A controlled trial of 2-month, 3-month, and 12-month regimens of chemotherapy for sputum smear-negative pulmonary tuberculosis: the results at 30 months. Am Rev Respir Dis 124: 138-142.

5. East and Central African-British Medical Research Council (1983) Controlled clinical trial of 4 short-couse regimens of chemotherapy (three 6-month and one 8-month) for pulmonary tuberculosis. Tuberc 64: 153-166.

6. East and Central African-British Medical Research Council (1986) Controlled clinical trial of 4 short-course regimens of chemotherapy (three 6-month and one 8-month) for pulmonary tuberculosis: final report. Tuberc 67: 5-15.

7. Jindani A, Nunn AJ, Enarson DA (2004) Two 8-month regimens of chemotherapy for treatment of newly diagnosed pulmonary tuberculosis: international multicentre randomised trial. Lancet 364: 1244-1251.

8. Felten MK (1989) Importance of rifampicin in combined daily/intermittent chemotherapy for tuberculosis. S Afr Med J 75: 524-526.

9. Singapore Tuberculosis Service-British Medical Research Council (1979) Clinical trial of six-month and four-month regimens of chemotherapy in the treatment of pulmonary tuberculosis. Am Rev Respir Dis 119: 579-585.

10. Singapore Tuberculosis Service-British Medical Research Council (1981) Clinical trial of six-month and four-month regimens of chemotherapy in the treatment of pulmonary tuberculosis: the results up to 30 months. Tuberc 62: 95-102.

11. Singapore Tuberculosis Service-British Medical Research Council (1986) Long-term follow-up of a clinical trial of six-month and four-month regimens of chemotherapy in the treatment of pulmonary tuberculosis. Am Rev Respir Dis 133: 779-783.

12. Sharifi-Mood B, Metanat M, Alavi-Naini R, Kouhpayeh HR, Salehi M, et al. (2006) The comparison of six-month and four-month regimens of chemotherapy in the treatment of smear positive pulmonary tuberculosis. J Med Sci 6: 108-111.

13. Poh SC, Singapore British Medical Research Council (1978) Controlled trial of 4-month and 6-month regimens of chemotherapy in the treatment of pulmonary tuberculosis. Annals of the Academy of Medicine 5: 242-243.

14. Hong Kong Chest Service-Tuberculosis Research Centre MBMRC (1991) A controlled clinical comparison of 6 and 8 months of antituberculosis chemotherapy in the treatment of patients with silicotuberculosis in Hong Kong. Am Rev Respir Dis 143: 262-267.

15. British Thoracic and Tuberculosis Association (1975) Short-course chemotherapy in pulmonary tuberculosis. Lancet 305: 119-124.

16. British Thoracic and Tuberculosis Association, Angel H (1976) Short-course chemotherapy in pulmonary tuberculosis. A controlled trial by the British Thoracic and Tuberculosis Association. Lancet 2: 1102-1104.

17. British Thoracic and Tuberculosis Association (1976) Short-course chemotherapy in pulmonary tuberculosis. Lancet 1102.

18. Perriens JH, St.Louis M, Mukadi YB, Brown C, Prignot J, et al. (1995) Pulmonary tuberculosis in HIV-infected patients in Zaire. N Engl J Med 332: 779-784.

19. Combs D, O'Brien R, Geiter LJ (1990) USPHS tuberculosis short-course chemotherapy trial 21: effectiveness, toxicity, and acceptability. Ann Intern Med 112: 397-406.

20. El-Sadr WM, Perlman DC, Matts JP, Nelson ET, Cohn DL, et al. (1998) Evaluation of an intensive intermittent-induction regimen and duration of short-course treatment for human immunodeficiency virus-related pulmonary tuberculosis. Clinical Infectious Diseases 26: 1148-1158.

21. Engbaek HC, Heckscher T, Hojgaard C, Larsen SO, Rasmussen KN, et al. (1981) Tuberculosis treated with rifampicin, ethambutol and isoniazid: Danish tuberculosis trial 1972-1974. Eur J Respir Dis 63: 84-93.

22. Howell F, O'Laoide R, Kelly P, ower J, lancy L (1989) Short course chemotherapy for pulmonary tuberculosis A randomised controlled trial of a six month versus a nine month oral regimen. Irish Medical Journal 82: 11-13.

23. British Thoracic Association (1981) A controlled trial of six months chemotherapy in pulmonary tuberculosis. First Report: results during chemotherapy. Br J Dis Chest 75: 141-153.

24. British Thoracic Association (1982) A controlled trial of six months chemotherapy in pulmonary tuberculosis. Second report: results during the 24 months after the end of chemotherapy. Am Rev Respir Dis 126: 460-462.

25. Zierski M, Bek E, Long MW, Snider DE Jr (1981) Short-course (6-month) cooperative tuberculosis study in Poland: results 30 months after completion of treatment. Am Rev Respir Dis 124: 249-251.

26. Hong Kong Chest Service-British Medical Research Council (1982) Controlled trial of 4 three-times-weekly regimens and a daily regimen all given for 6 months for pulmonary tuberculosis. Second report: the results up to 24 months. Tuberc 63: 89-98.

27. Hong Kong Chest Service-British Medical Research Council (1987) Five-year follow-up of a controlled trial of five 6-month regimens of chemotherapy for pulmonary tuberculosis. Am Rev Respir Dis 136: 1339-1342.

28. Teo SK, Tan KK, Khoo TK (2002) Four-month chemotherapy in the treatment of smear-negative pulmonary tuberculosis: results at 30 to 60 months. Ann Acad Med Singapore 31: 175-181.

29. Macnab MF, BohmerPD, Seager JR (1994) Evaluation of the 3-drug combination, Rifater, versus 4-drug therapy in the ambulatory treatment of tuberculosis in Cape Town. S Afr Med J 84: 325-327.

30. East African-British Medical Research Councils (1974) Controlled clinical trial of four short-course (6-month) regimens of chemotherapy for treatment of pulmonary tuberculosis. Lancet 304: 1100-1106.

31. East African-British Medical Research Council (1976) Controlled clinical trial of four (6-month) 3 regimens of chemotherapy for pulmonary tuberculosis. Second report. Am Rev Respir Dis 114: 471-475.

32. Hong Kong Chest Service and British Medical Research Council (1978) Controlled trial of 6-month and 8-month regimens in the treatment of pulmonary tuberculosis. First report. Am Rev Respir Dis 118: 219-228.

33. Hong Kong Chest Service and British Medical Research Council (1979) Controlled trial of 6-month and 8-month regimens in the treatment of pulmonary tuberculosis: the results up to 24 months. Tuberc 60: 201-210.

34. Third East African-British Medical Research Councils (1978) Controlled clinical trial of four short-course regimens of chemotherapy for two durations in the treatment of pulmonary tuberculosis: first report. Am Rev Respir Dis 118: 39-48.

35. Third East African-British Medical Research Council (1980) Controlled clinical trial of four short-course regimens of chemotherapy for two durations in the treatment of pulmonary tuberculosis. Second report. Tuberc 61: 59-69.

36. Balasubramanian R, Sivasubramanian S, Vijayan VK, Ramachandran R, Jawahar MS, et al. (1990) Five year results of a 3-month and two 5-month regimens for the treatment of sputum-positive pulmonary tuberculosis in south India. Tuberc 71: 253-258.

37. Tuberculosis Research Centre Madras (1986) A controlled clinical trial of 3- and 5-month regimens in the treatment of sputum-positive pulmonary tuberculosis in South India. Am Rev Respir Dis 134: 27-33.

38. Long MW, Snider JrD, Farer LS (1979) U.S. Public health service cooperative trial of three rifampin-isoniazid regimens in treatment of pulmonary tuberculosis. Am Rev Resp Dis 119: 879-894.

39. Corpe R (1972) Rifampin in original treatment of pulmonary tuberculosis A United States public health service cooperative therapy trial. Bull Int Union Tuberc 47: 41-47.

40. Snider Jr.DE, Long MW, Cross FS, Farer LS (1984) Six-months isoniazid-rifampin therapy for pulmonary tuberculosis. Am Rev Respir Dis 129: 573-579.

41. Tripathy SP (1979) Madras study of short-course chemotherapy in pulmonary tuberculosis. Bull Int Union Tuberc 54: 28-30.

42. Tuberculosis Research Centre (1983) Study of chemotherapy regimens of 5 and 7 months' duration and the role of corticosteroids in the treatment of sputum-positive patients with pulmonary tuberculosis in South India. Tuberc 64: 73-91.

43. Santha T, Nazareth O, Krishnamurthy M S, Balasubramanian R, Vijayan V K, et al. (1989) Treatment of pulmonary tuberculosis with short course chemotherapy in south India-5 year follow up. Tuberc 70: 229-234.

44. Snider DE, Gracyk J, Bek E, Rogowski J (1984) Supervised Six-months treatment of newly diagnosed pulmonary tuberculosis using isoniazid, rifampin, and pyrazinamide with and without streptomycin. Am Rev Respir Dis 130: 1091-1094.

45. Singapore Tuberculosis Service-British Medical Research Council (1985) Clinical trial of three 6-month regimens of chemotherapy given intermittently in the continuation phase in the treatment of pulmonary tuberculosis. Am Rev Respir Dis 132: 374-378.

46. Singapore Tuberculosis Service British Medical Research Council (1988) Five-year follow-up of a clinical trial of three 6-month regimens of chemotherapy given intermittently in the continuation phase in the treatment of pulmonary tuberculosis. Am Rev Respir Dis 137: 1147-1150.

47. Mazouni L, Tazir M, Boulahbal F, Chaulet P (1985) Enquête contrôlée comparant trois règimes de chiniothérapie quotidienne de six mois dans la tuberculose pulmonaire, en pratique de routine à Alger. Rev Mal Resp 2: 209-214.

48. Kohno S, Koga H, aku M, aesaki S, ara K (1992) Prospective Comparative Study of Ofloxacin or Ethambutol for the Treatment of Pulmonary Tuberculosis. Chest 102: 1815-1818.

49. Teo SK (1999) Assessment of a combined preparation of isoniazid, rifampicin and pyrazinamide (Rifater) in the initial phase of chemotherapy in three 6-month regimens for smear-positive pulmonary tuberculosis: a five-year follow-up report. Int J Tuber Lung Dis 3: 126-132.

50. Hong Kong Chest-British Medical Research Council (1981) Controlled trial of four twice-weekly regimens and a daily regimen all given for 6 months for pulmonary tuberculosis. Lancet 317: 171-174.

51. Kennedy N, Berger L, Curram J, Fox R (1996) Randomized controlled trial of a drug regimen that includes ciprofloxacin for the treatment of pulmonary tuberculosis. Clinical Infectious Diseases 22: 827-833.

52. Algerian Working Group British Medical Research Council Cooperative Study (1991) Short-course Chemotherapy for Pulmonary Tuberculosis under Routine Programme Conditions: a comparison of regimens of 28 and 36 weeks duration in Algeria. Tuberc 72: 88-100.

53. Hong Kong Chest Service-British Medical Research Council (1991) Controlled trial of 2, 4, and 6 months of pyrazinamide in 6-month, three-times-weekly regimens for smear-positive pulmonary tuberculosis, including an assessment of a combined preparation of isoniazid, rifampin, and pyrazinamide. Results at 30 months. Am Rev Respir Dis 143: 700-706.

54. Singapore Tuberculosis Service-British Medical Research Council (1991) Assessment of a daily combined preparation of isoniazid, rifampin, and pyrazinamide in a controlled trial of three 6-month regimens for smear-positive pulmonary tuberculosis. Am Rev Respir Dis 143: 707-712.

55. Chaulet P, Boulahbal F (1995) Essai clinique d'une combinaison en proportions fixes de trois medicaments dans le traitement de la tuberculose. Tuberc Lung Dis 76: 407-412.

56. Su W-J, Perng R-P (2002) Fixed-dose combination chemotherapy (Rifater/Rifinah) for active pulmonary tuberculosis in Taiwan: a two-year follow-up. Int J Tuberc Lung Dis 6: 1029-1032.

57. Pedral-Sampaio DB, Netto EM, Brites C, Bandeira AC, Guerra C, et al. (2003) Use of Rhu-GM-CFS in pulmonary tuberculosis patients: results of a randomized clinical trial. Brazilian Journal of Infectious Diseases 7: 245-252.

58. East African-British Medical Research Council Study (1972) Controlled clinical trial of short-course (6 month) regimens of chemotherapy for treatment of pulmonary tuberclosis. Lancet 299: 1079-1085.

59. East African-British Medical Research Councils (1973) Controlled clinical trial of four short-course (6-month) regimens of chemotherapy for treatment of pulmonary tuberculosis. Second report. Lancet 1: 1331-1338.

60. East African-British Medical Research Councils (1974) Controlled clinical trial of four short-course (6-month) regimens of chemotherapy for treatment of pulmonary tuberculosis. Third report. Lancet 2: 237-240.

61. British Medical Research Council (1973) Co-operative controlled trial of a standard regimen of streptomycin, PAS and isoniazid and three alternative regimens of chemotherapy in Britain. Tuberc 54: 99-129.

62. Poppe de Figeuiredo F, Alves Brito A, Laborne Valle JH, Martins Tavares P, Linhares Trannin P (1974) Short duration chemotherapy of pulmonary tuberculosis: a pilot trial. Bull Int Union Against Tuberculosis 49: 382.

63. Singapore Tuberculosis Service, British Medical Research Council (1975) Controlled trial of intermittent regimens of rifampicin plus isoniazid for pulmonary tuberculosis in Singapore. Lancet 306: 1105-1109.

64. Singapore Tuberculosis Service-British Medical Research Council (1977) Controlled trial of intermittent regimens of rifampin plus isoniazid for pulmonary tuberculosis in Singapore. The results up to 30 months. Am Rev Respir Dis 116: 807-820.

65. Shenna DH (1984) Comparison of a conventional and an initial 2-month intensive drug regimen for treating pulmonary tuberculosis in Swaziland. Tuberc 65: 101-104.

66. Tripathy SP (1983) Controlled clinical trial of a 3-month and two 5-month regimens in pulmonary tuberculosis. Second Madras short course study. Bull Int Union Tuberc 58: 97-100.

67. Algerian working group-British Medical Research Council (1984) Controlled clinical trial comparing a 6-month and a 12-month regimen in the treatment of pulmonary tuberculosis in the Algerian Sahara. Am Rev Respir Dis 129: 921-928.

68. Tanzania-British Medical Research Council (1985) Controlled clinical trial of two 6-month regimens of chemotherapy in the treatment of pulmonary tuberculosis. Am Rev Respir Dis 131: 727-731.

69. Hong Kong Chest Service-Tuberculosis Research Centre Madras-British Medical Research Council (1989) A controlled trial of 3-month, 4-month, and 6-month regimens of chemotherapy for sputum-smear-negative pulmonary tuberculosis. Results at 5 years. Am Rev Respir Dis 139: 871-876.

70. Kenyan-Zambian-British Medical Research Council (1989) Controlled clinical trial of levamisole in short-course chemotherapy for pulmonary tuberculosis. Am Rev Respir Dis 140: 990-995.

71. Tam CM, Chan SL, Lam CW, Leung CC, Kam KM, et al. (1998) Rifapentine and isoniazid in the continuation phase of treating pulmonary tuberculosis. Am J Respir Crit Care Med 157: 1726-1733.

72. Murray J, Sonnenberg P, Shearer S, Godfrey-Faussett P (2000) Drug-resistant pulmonary tuberculosis in a cohort of southern African goldminers with a high prevalence of HIV infection. S Afr Med J 90: 381-386.

73. Agounitestane D, Chiheb M, Khaled S, Khaled NA, Boulahbal F, et al. (1990) Essai thérapeutique d'une combinaison de trois médicaments essentiels dans la chimiothérapie courte de la tuberculose. Rev Mal Resp 7: 209-213.

74. Mohanty KC, Dhamgaye TM (1993) Controlled of ciprofloxacin in short-term chemotherapy for pulmonary tuberculosis. Chest 104: 1194-1198.

75. Zhang LX, Kan GQ, Tu DH, Wan LY, Faruqi AR (1996) Fixed-dose combination chemotherapy versus multiple, single-drug chemotherapy for tuberculosis. Current Therapeutic Research 57: 849-856.

76. Gonzalez-Montaner LJ, Natal S, Yongchaiyud P, Olliaro P, the Rifabutin Study Group (1994) Rifabutin for the treatment of newly-diagnosed pulmonary tuberculosis: a multinational, randomized, comparative study versus Rifampicin. Tuberc Lung Dis 75: 341-347.

77. Maureen M, McGregor M, Olliaro P, Wolmarans L, Mabuza B, et al. (1996) Efficacy and safety of rifabutin in the treatment of patients with newly diagnosed pulmonary tuberculosis. Am J Respir Crit Care Med 154: 1462-1467.
